# Genomic Survey of Salt Acclimation-Related Genes in the Halophilic Cyanobacterium *Euhalothece* sp. Z-M001

**DOI:** 10.1038/s41598-020-57546-1

**Published:** 2020-01-20

**Authors:** Hee Wook Yang, Ji Young Song, Sung Mi Cho, Hak Cheol Kwon, Cheol-Ho Pan, Youn-Il Park

**Affiliations:** 1 Department of Biological Sciences, Chungnam National University, Daejeon, 34134 Korea; 2 Natural Product Informatics Research Center, KIST Gangneung Institute of Natural Products, Gangneung, 25451 Korea; 3 Present Address: Unit of Polar Genomics, Korea Polar Research Institute, Incheon, 21990 Korea

**Keywords:** Photosynthesis, Plant molecular biology, Plant physiology, Plant stress responses, Secondary metabolism, Bacteria, Microbiology, Plant sciences

## Abstract

Like other halophilic cyanobacterial genomes, the *de novo*-assembled genome of *Euhalothece* sp. Z-M001 lacks genes encoding keto-carotenoid biosynthesis enzymes, despite the presence of genes encoding carotenoid-binding proteins (CBPs). Consistent with this, HPLC analysis of carotenoids identified β-carotene and zeaxanthin as the dominant carotenoids. CBPs coexpressed with the zeaxanthin biosynthesis gene increased the survival rates of *Escherichia coli* strains by preventing antibiotic-induced accumulation of reactive oxygen species (ROS). RNA-seq analysis of *Euhalothece* revealed that among various salt resistance-related genes, those encoding the Na^+^ transporting multiple resistance and pH adaptation (Mrp) systems, glycine betaine biosynthesis enzymes, exopolysaccharide metabolic enzymes, and CBPs were highly upregulated, suggesting their importance in hypersaline habitats. During the early phase of salt deprivation, the amounts of β-carotene and zeaxanthin showed a negative correlation with ROS content. Overall, we propose that in some halophilic cyanobacteria, β-carotene and zeaxanthin, rather than keto-carotenoids, serve as the major chromophores for CBPs, which in turn act as effective antioxidants.

## Introduction

Cyanobacteria are photosynthetic prokaryotes that exhibit diverse protective mechanisms to cope with harsh environmental conditions. One of the protective mechanisms involves the use of carotenoids^1^ and diverse carotenoid-binding proteins (CBPs)^2^, which play essential roles in protecting the photosynthetic apparatus from damage by both excess light energy and reactive oxygen species (ROS). Most cyanobacterial CBPs bind to β-carotene (β-Car) and its oxygenated derivatives, called xanthophylls (Xan), and are located in photosynthetic protein complexes such as photosystem I (PSI), PSII, and cytochrome *b*_6_ *f* ^3^. By contrast, water soluble CBPs bind non-covalently to cyanobacterial keto-carotenoids, such as echinenone (Ech) and canthaxanthin (Can), and to glycosylated-carotenoids such as myxoxanthophyll (Myx)^4^; CBPs then function as effective energy dissipaters by interacting with the light-harvesting antenna, phycobilisome (PBS)^5^. CBPs also bind to zeaxanthin (Zea), albeit with low affinity^6^.

The orange carotenoid protein (OCP) and its two paralogs, helical carotenoid protein (HCP) and C-terminal domain homolog (CTDH), are the only known soluble CBPs^4^. The OCP is composed of two globular domains (one each at the N- and C-terminal ends); a single carotenoid is embedded between the two domains via a hydrophobic interaction^7^. Blue light absorption by a keto-carotenoid results in a conformational change in the OCP, from the orange inactive form (OCP°) to the red active form (OCP^R^). Binding of OCP^R^ to PBS quenches PBS fluorescence, which is prevented by fluorescence recovery protein (FRP)^8^. FRP prevents accumulation of the OCP^R^ by interacting with the C-terminal domain of OCP^8^. The biological functions of HCPs, which are grouped into nine distinct clades^7^, are largely unknown. HCP4 isolated from the cyanobacterium *Anabaena* PCC 7120 binds to PBS and dissipates excess light as heat, whereas HCP2 and HCP3 quench O_2_^9^. In *Nostoc flagelliforme*, these biochemical functions are related to dehydration resistance^10^. By contrast, CTDH transfers a single keto-carotenoid to a nearby HCP^11^.

Cyanobacteria are divided into two major groups, euryhaline and stenohaline, based on their salt tolerance levels^12^. *Synechocystis* PCC6803 (hereafter referred to as *Synechocystis*) is a euryhaline cyanobacterium capable of adapting to a wide range of salinity, from fresh water to 1.2 M NaCl^13^. Stenohaline strains viable within a narrow range of salinity include the *Halothece* cluster from hypersaline environments^14^; however, many of their physiochemical traits and protective mechanisms that operate under low-salt stress remain unknown. Generally, salt stress negatively affects the growth rate of algae. To cope with salt stress, cyanobacteria prevent entry of Na^+^ ions into the cell using Na^+^/H^+^ antiporters and K^+^ transporters, or by accumulating various compatible solutes such as sucrose, trehalose, glucosylglycerol, proline, and glycine betaine^15^. Additionally, exopolysaccharides (EPSs) protect cyanobacteria against osmotic/salt stress^16^. Direct experimental evidence supporting the protective role of EPSs against various heavy metals^17,18^ is available for the freshwater unicellular cyanobacterium *Synechocystis*.

Interestingly, despite the presence of CBPs, some stenohaline cyanobacteria, including *Dactylococcopsis salina* PCC8305 and *Microcoleus* sp. IPPAS B-353, lack genes encoding keto-carotenoids. Moreover, cyanobacteria such as *Prochlorococcus* sp. lack not only genes encoding keto-carotenoids but also genes encoding CBPs (Supplementary Table S1). Based on publicly available genome information and *de novo* genomic data from *Euhalothece* sp. Z-M001 (hereafter referred to as *Euhalothece*), we found that some haloalkaliphilic cyanobacterial species, including *Euhalothece*, lack the gene encoding keto-carotenoids but harbor CBP-encoding genes. This finding raises an interesting question about the nature and physiological functions of chromophores of water soluble CBPs in cyanobacteria lacking the ketolase gene. Furthermore, the expression profiles of genes involved in salt resistance via extrusion of Na^+^ ions, biosynthesis of compatible compounds, production of EPSs, and transcription of heat shock proteins^15,19^ are largely unknown in this cyanobacterium.

*Euhalothece* is a single-celled, stenohaline cyanobacterium (Supplementary Table S1) requiring 7% NaCl for optimal growth. It exhibits considerable morphological variability depending on the pH, and on the concentrations of NaCl and carbonate^20^. Here, we investigated prevailing salt stress resistance strategies adopted by *Euhalothece*. Like *Dactylococcopsis salina* PCC8305, the genome of the *Euhalothece* (BioProject DB ID: PRJNA557809; http://168.188.109.191/cyanomain/) harbors genes encoding OCP, HCP, and CTDH, but lacks genes related to keto-carotenoid biosynthesis. Using transcriptomic approaches, we found that transcript levels of genes encoding EPS biosynthesis enzymes and soluble CBPs vary according to salt concentration. We then isolated genes encoding carotenoid biosynthesis proteins and CBPs. Functional characterization of these recombinant proteins suggests that CBPs function as antioxidants during salt deprivation.

## Results and Discussion

### Phylogenetic analysis and genome of *Euhalothece*

Phylogenetic analysis of 16S rRNA sequences derived from 59 cyanobacterial species, three eubacteria (used as outgroups)^21,22^, and three additional species (*Euhalothece* Z-M001, *Euhalothece* Z9404, and *Nostoc punctiforme* ATCC29133) revealed that *Euhalothece* is closely related to the stenohaline cyanobacterium *Dactylococcopsis salina* PCC8305 (supported by a perfect bootstrap value) (Supplementary Fig. S1; red color). This clade does not contain the *Microcoleus* and *Geitlerinema* taxa that form the basis of a monophyletic group comprising freshwater cyanobacteria subsections I and II. Current phylogeny reconstruction indicates that the halophilic cyanobacteria group is associated with diversification of freshwater cyanobacteria.

The complete circular genome of *Euhalothece* (GenBank ID: CP042326) is 3,317,848 bp in length (average GC content = 41.03%) and contains 3,354 coding sequences (CDSs), six rRNAs, 43 tRNAs, and three clustered regularly interspaced short palindromic repeat arrays (Supplementary Fig. S2). Genes responsible for carotenoid biosynthesis, including *crtR* (*ezr1976*) and *cruA* (*ezl1782*), and CBPs such as *ocp* (*ezr1969*), *hcp* (*ezr2074*), and *ctdh* (*ezr2075*), were distributed on both strands, and no strong GC skew or single DNA replication origin inflection point was detected. In addition to the main chromosome, four scaffolds (GenBank IDs: CP042327–CP042330) were assembled into plasmids. All plasmids were circular, varying in size (14,665–67,514 bp), G + C content (40.04–40.85%), and gene number (20–83). In plasmids, genes encoding mobile element proteins, ParAB, primase, DNA invertase, DNA integrase, transposase, and phage tail protein, were identified, but those encoding CBPs and proteins involved in carotenoid biosynthesis and EPS metabolism were absent. Overall, the four plasmids were different but contained a 1.6 kb long block of similar sequence; the remaining plasmid sequence shared no similarity.

### Growth, photosynthetic pigments, and EPS content of *Euhalothece* acclimated to salt-depleted and -repleted conditions

**Figure 1:**
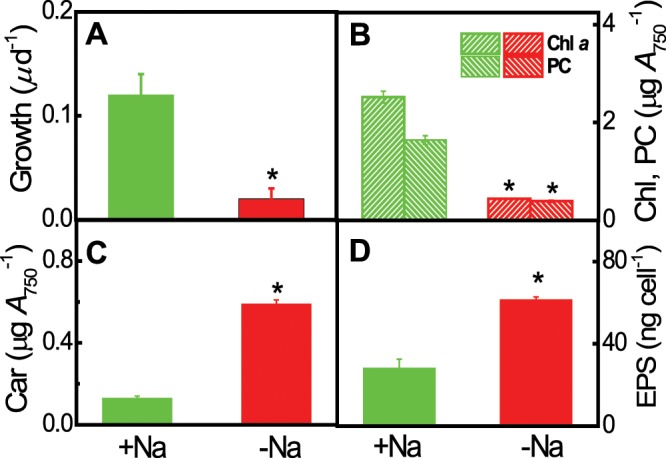
Growth, photosynthesis pigments, and exopolysaccharide (EPS, D) content in *Euhalothece* sp. Z-M001 maintained for 6 days on S growth medium containing 0% or 3% NaCl. (**A**–**D**) Growth (**A**), chlorophyll *a* (Chl) and phycocyanin (PC) content (**B**), carotenoid (Car) content (**C**), and exopolysaccharide (EPS) content (**D**) of *Euhalothece* cells. Cells grown in 3% NaCl-containing liquid S-medium (+Na) were transferred to 0% NaCl (−Na) medium under growth light conditions for 6 days. Data represent mean ± S.E. (*n* = 3–5). Significant differences are indicated with an asterisk (*p < 0.05; Student’s *t*-test).

In this study, salt deprivation retarded the growth of *Euhalothece* cells. Cells grown for 6 days on medium containing 3% NaCl grew faster (Fig. 1A), and contained more Chl *a* and phycocyanin (PC) than those grown under the salt-deprived conditions (Fig. 1B). By contrast, the total carotenoid and EPSs contents of cells under salt-depleted conditions increased significantly by several-fold (Fig. 1C) and 2.1-fold (Fig. 1D), respectively. Thus, *Euhalothece* seems to adopt primarily EPS-based salt-out and carotenoid-based ROS scavenging strategies under varying external salt concentrations.

### Salt acclimation genes associated with ion homeostasis, compatible solutes, EPS biosynthesis, and two component systems in *Euhalothece*

**Figure 2:**
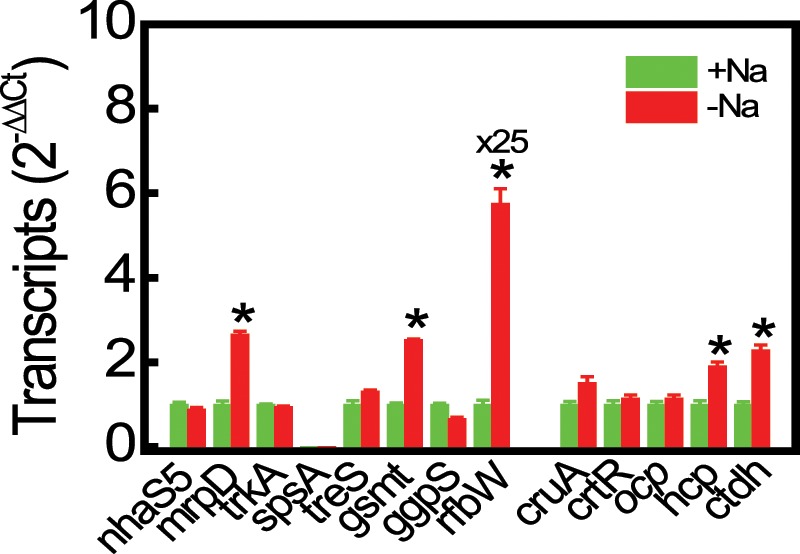
Quantitative real-time PCR (qRT-PCR) analysis of representative salt resistance-related genes in *Euhalothece* Z-M001 maintained for 6 days on S growth medium containing 0% (−Na)or 3% NaCl (+Na). *nhaS5* (*ezl1331*; Na^+^/H^+^ antiporter), *mrpD* (*ezr1945*; multisubunit Na^+^/H^+^ antiporter), *trkA* (*ezl0593*; K^+^ uptake protein), *spsA* (*ezl3057*; sucrose phosphate synthase), *treS* (*ezr3241*; trehalose synthase), *gsmt* (*ezr2095*; glycine-sarcosine-N-methyltransferase), *ggpS* (*ezr0090*; glucosylglycerol-phosphate synthase), *rfbW* (*EZL0736*; mannosyl transferase), *cruA* (*ezl1782*; lycopene cyclase), *crtR* (*ezr1976*; β-carotene hydroxylase), *ocp* (*ezr1969*; orange carotenoid protein), *hcp* (*ezr2074*; helical carotenoid protein), and *ctdh* (*ezr2075*; C-terminal domain homolog). Data represent the mean ± S.E. (*n* = 3–5). Significant differences are indicated by an asterisk (*p < 0.05; Student’s *t*-test).

Monovalent cation transporters such as Na^+^/H^+^ antiporters (NhaS1–S6), multisubunit Na^+^/H^+^ antiporters (MrpA–G), K^+^ transporters (KtrA/B and KdpA–D), and K^+^ uptake protein (TrkA) are involved in salt acclimation^15,19^. The newly sequenced *de novo*-assembled genome of *Euhalothece* harbors genes encoding these cation transporters. The *Euhaothece* genome contains 12 genes encoding Na^+^ pumps, including six *nhaS* genes and six multiple resistance and pH adaptation (Mrp) system genes (*mrpB/C/D/E/F/G*) (Supplementary Table S2). The high-affinity ATP-dependent K^+^ transport system consists of KdpABC(D) subunits and is found in some cyanobacteria such as *Nostoc* 7120 and *Synechocystis*^19^. However, as in the majority of marine picoplanktonic strains, this Kdp system was not detected in the *Euhalothece* genome. By contrast, the KtrAB(E) system, which is present in all known cyanobacterial genomes and presumably plays a key role in K^+^ import^19,23^, was identified in the genome of *Euhalothece* (Supplementary Table S2). Among various transporter-encoding genes, the *mrp* gene clusters seem to play a major role in *Euhalothece*, since genes encoding these multisubunit Na^+^/H^+^ antiporters were constitutively expressed under salt-deficient (0% NaCl) and -sufficient (3% NaCl) conditions, as revealed by RNA-seq profiling (Supplementary Table S2) and qRT-PCR analysis (Fig. 2).

Among compatible solutes such as sucrose, trehalose, glucosylglycerol, and glycine betaine, glucosylglycerate, and proline, genes encoding proteins involved in biosynthesis of sucrose (*spsA* and *spp*), trehalose (*treS* and *treY*), glucosylglycerol (*ggpP* and *ggpS*), glycine betaine (*gsmt* and *sdmt*), and proline (*proA/B/C*) were annotated; however, genes encoding glucosylglycerate biosynthesis proteins were absent from the *Euhalothece* genome. Transcripts of genes encoding GSMT and SDMT were highly abundant compared with those of genes involved in biosynthesis of other solutes (Supplementary Table S2, Fig. 2). This suggests that glycine betaine acts a major compatible solute that protects against salt stress in *Euhalothece*, as in other hypersaline cyanobacteria^24^.

EPSs are anionic due to the presence of uronate and sulphate groups, which chelate cations such as Na^+^ and heavy metal ions in various photosynthetic and non-photosynthetic bacteria. Over 500 putative genes associated with the cell surface structure have been identified in *Synechocystis* based on their homology to genes in *E. coli* and *Salmonella*^25^. To date, a few dozen putative genes have been tested experimentally. The gene cluster *sll1722–sll1726*, presumed to encode glycosyltransferases^26^, and the *exoD* (*Slr1875*), *gumB* (*Sll1581*), and *gumC* (*Sll0923*) genes^27^ are involved in production of EPSs and in protection against various stresses caused by light, H_2_O_2_, NaCl, CdSO_4_, CoCl_2_, and Fe starvation. Furthermore, *Synechocystis* genes encoding permeases (*slr0977* and *sll0574*) and ABC-transporters (*slr0982* and *sll0575*) are involved in production of EPSs, which in turn play a crucial role in cell adherence^25^. Among the 151 *Synechocystis* gene homologs^25^, 118 genes potentially involved in polysaccharide biosynthesis were identified in the genome of *Euhalothece* (Supplementary Table S2). These 118 genes were expressed at different levels in the presence of 3% NaCl, but only six were responsive to salt deprivation. Expression of five genes, including *spsA*, *epsB*, *rfbW*, *hlyB*, and *rfbP*, increased by more than 2-fold, while that of only one gene encoding glucosyl transferase fell (Supplementary Table S2 and Fig. 2). Thus, *Euhalothece* utilizes EPS production as one salt acclimation strategy for survival in hypersaline natural habitats.

The K^+^ transporter system Ktr^23^ and the glucosylglycerol biosynthesis protein GgpS^28^ in *Synechocystis* are regulated directly by the intracellular salt concentration. In addition to the post-translational regulatory mechanism, ion content directly regulates expression of salt-responsive genes at the transcriptional level via alternative sigma factor-dependent changes in the promoter selectivity of RNA polymerase^29,30^. In addition to sigma factor cascades, two component systems comprising a sensory histidine kinase (Hik) and the corresponding response regulator (Rre) mediate salt responses. For instance, Hik proteins such as Hik16, Hik33, Hik34, and Hik41^29^, along with Rre proteins^31,32^, act as salt sensors in *Synechocystis*. The genome of *Euhalothece* encodes two component systems comprising 41 Hik and 41 Rre proteins. Among the 41 Hik-encoding genes, expression of only three genes (*Ezl3071*, *Ezr1858*, and *Ezr2811*) was up- or down-regulated in response to a change in salt concentration (Supplementary Table S2). Thus, these Hik proteins could act as sensors of varying salt concentrations, although further investigation is needed.

### *crt*s and *cbp*s in the *Euhalothece* genome

**Figure 3:**
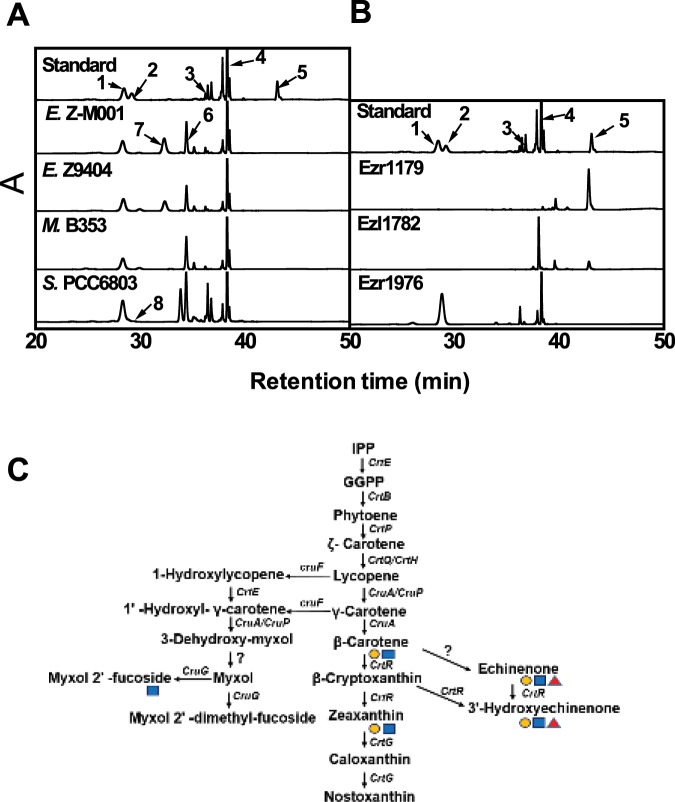
Carotenoid composition of halophilic cyanobacteria and *E. coli* strains containing *Euhalothece* genes required for lycopene and zeaxanthin biosynthesis, and a proposed carotenoid biosynthetic pathway. (**A**) High-performance liquid chromatography (HPLC) analysis of carotenoid profiles in *Euhalothece* Z-M001, *Euhalothece* Z9404, *Microcoleus* B353, and *Synechocystis* PCC 6803. 1, Zeaxanthin; 2, Canthaxanthin; 3, Echinenone; 4, β-carotene; 5, Lycopene; 6, Chlorophyll; 7, *Cis*-Zeaxanthin; 8, 3’-hydroxyechinenone. (**B**) Carotenoid extracts prepared from *E. coli* cells harboring PACtrcp-LYC (for lycopene synthesis) and either pBAD-Ezr1179 or pBAD-Ezl1782 (for β-carotene synthesis), and from *E. coli* harboring PACtrcp (for β-carotene synthesis) and PACtrcp-Ezr1976 (for zeaxanthin synthesis). (**C**) Proposed carotenoid biosynthesis pathway in *Euhalothece* Z-M001. Orange circle, OCP; blue square, HCP; red triangle, CTDH.

In addition to the above-mentioned genes involved in salt acclimation via regulation of intracellular water potential, carotenoids play protective roles against salt stress by promoting protein and membrane stabilization, and by scavenging ROS; this occurs, for example, in the mesophilic cyanobacterium *Synechocystis*^30^. Thus, the carotenoid composition of *Euhalothece* was determined along with that of other halophilic cyanobacteria such as *Euhalothece* Z9404 and *Microcoleus* B353 (Fig. 3A). These three cyanobacterial species are halophilic and lack keto-carotenoid biosynthesis genes such as *crtW* and *crtO*; this is also true for other halophilic cyanobacteria such as *Halothece* PCC7418 and *Dactylococcopsis salina* PCC8305 (Supplementary Fig. S1).

HPLC analysis detected trace amounts of Ech, but not Can, in these three stenohaline cyanobacteria, whereas Ech was identified as a dominant carotenoid in *Synechocystis* (Fig. 3A). In addition, Zea (5–6% of the total carotenoids) and β-Car (11–13% of the total carotenoids) were detected in *Euhalothece* and two other stenohaline cyanobacteria. A peak representing unknown compounds was detected in *Euhalothece*, the retention time of which was less than that of Can and Zea but more than that of Ech. This implies that the compound in the unknown peak is either more hydrophobic than both Can and Zea, but more hydrophilic than Ech, or a structurally different isoform (i.e., the *cis*-form) of the same carotenoid^14^.

A search for homologs in the complete genome sequence of *Euhalothece* using the functionally confirmed carotenogenesis genes of both *Synechocystis* and *Nostoc* sp. PCC 7120^33,34^ as query sequences revealed the presence of *crtE*, *crtB*, *crtP*, *crtQ*, *crtH*, *cruA*, *cruP*, *cruG*, and *crtR* genes in *Euhalothece*. Among these genes, *Ezl1782* and *Ezr1976* were designated as genes encoding lycopene cyclase (*cruA*) and β-Car hydroxylase (*crtR*), respectively, because *E. coli* cells carrying these genes produced major peaks corresponding to β*-*Car and Zea, indicating that *Ezl1782* and *Ezr1976* gene products are involved in lycopene cyclization and β-Car hydroxylation in *Euhalothece* (Fig. 3B). Both genes were expressed constitutively in the presence of 3% NaCl and in the absence of NaCl (Supplementary Table S2, Fig. 2). However, as in several halotolerant and halophilic cyanobacterial genomes including *Microcoleus* sp. B353^22^ and *Halothece* sp. PCC7418^20^, no keto-carotenoid biosynthesis genes such as *crtW* and *crtO* were detected. The failure of several *Euhalothece crtW/O* candidate genes to produce keto-carotenoids in β*-*Car-accumulating *E. coli* is consistent with this genomic survey. Based on this information, we propose the carotenoid biosynthesis pathway present in *Euhalothece*^35,36^ (Fig. 3C). However, the absence of *crtW* and *crtO* does not necessarily equate to non-production of keto-carotenoids; indeed, it is consistent with previous studies showing that some halotolerant unicellular cyanobacteria, such as *Dactylococcopsis salina* PCC8305 and *Halothece* sp. PCC7418, harbor Ech as the predominant carotenoid^14^, despite the absence of *crtO* and *crtW* genes.

### EuCBPs bind to β-car, Zea, and keto-carotenoids

Genes with a predicted amino acid sequence similar to that of *crtW/O* were not identified in *Euhalothece*, but genes encoding OCP and its paralogs HCP and CTDH were present. The *Euhcp* gene was adjacent to the *Euctdh* gene. A phylogenetic tree of OCP, HCP, and CTDH constructed using the maximum likelihood method revealed that EuOCP belongs to the OCP1 clade (Supplementary Fig. S3A), which includes the well-studied OCPs from *Synechocystis* (SyOCP) and *Nostoc* sp. PCC7120 (NoOCP)^37^. In general, the *ocp1* gene co-exists with the *fluorescence recovery protein* (*frp*) gene in the genomes of most cyanobacteria^37^. However, the *frp* gene was absent from the genome of *Euhalothece*. The phylogenetic tree for HCP revealed that EuHCP belonged to the HCP5 clade (Supplementary Fig. S3B). EuCTDH clustered with proteins in one of the major CTDH clades (CTDH1) and showed 91% identity to the amino acid sequences of these proteins (Supplementary Fig. S3C).

**Figure 4:**
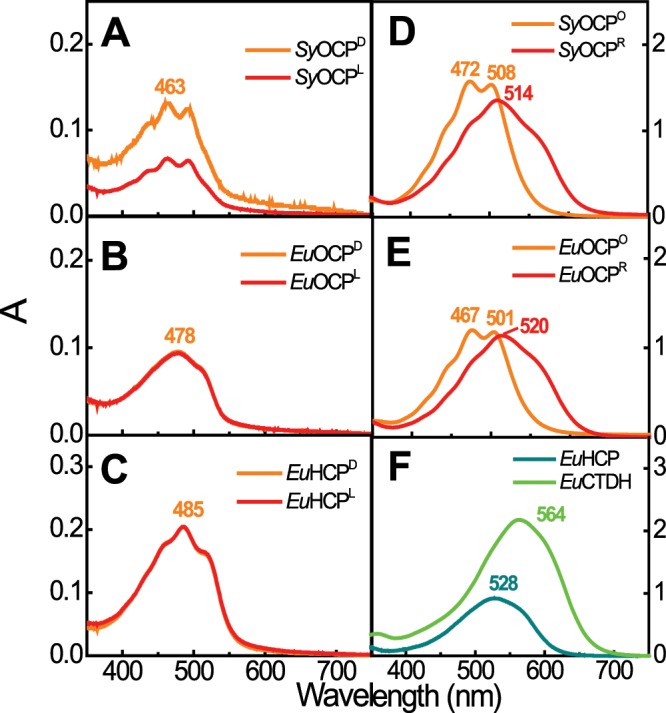
Absorption spectra of OCP and its paralogs, HCP and CTDH, purified from zeaxanthin (Zea)- and cantaxanthin (Can)-producing *E. coli*. (**A**–**F**) Absorption spectra of proteins purified from Zea-producing *E. coli* (**A**–**C**) and Can-producing *E. coli* (**D**–**F**). SyOCP, *Synechocystis* OCP (Slr0088); EuOCP, *Euhalothece* OCP (Ezr1969); EuHCP, *Euhalothece* HCP (Ezr2074); EuCTDH, *Euhalothece* CTDH (Ezr2075). Orange and red lines (**A**–**E**) represent absorption spectra of OCP and HCPs before (OCP^D^, OCP° and HCP^D^) and after exposure to light (OCP^L^, OCP^R^ and HCP^L^). Both EuHCP and EuCTDH bound to Can were non-responsive to illumination (**F**).

To test whether soluble EuCBPs bind to Zea, the *Euocp*, *Euhcp*, and *Euctdh* genes were expressed in Zea-producing *E. coli*. *Syocp* was also expressed in *E. coli* as a positive control. Recombinant SyOCP proteins bound to Zea but did not exhibit photoconversion activity upon blue light exposure (Fig. 4A)^6^. Consistent with this, recombinant EuOCP and EuHCP purified from Zea-producing *E. coli* failed to show a shift toward red light upon blue light illumination (Fig. 4B,C). Carotenoids were extracted from these holo-EuCBPs and analyzed by HPLC. Based on the retention times, carotenoids in the peaks were identified as Zea and β*-Car*, respectively (Supplementary Fig. S4).

To test whether EuCBPs bind to keto-carotenoids, the *Euocp*, *Euhcp*, and *Euctdh* genes were expressed in Can-producing *E. coli*. To verify that the EuCBPs expressed in Can-accumulating *E. coli* are photoactive, we measured the absorption spectra of purified holo-EuCBPs (Fig. 4E,F) and holo-SyOCP (Fig. 4D). A shift in the spectrum of the isolated EuOCP toward red light (active OCP) was detected upon exposure to blue light but, like SyOCP, active OCP reverted back to the inactive form in darkness. The maxima of the absorption spectra of both active and inactive EuOCP were slightly different from that of SyOCP. However, the time involved in the conversion from the active to inactive state, and the efficiency of chromophorylation (A_472_/A_280_ vs. A_467_/A_280_), were almost identical between EuOCP and SyOCP. This implies that the function of EuOCP is similar to that of SyOCP in the presence of keto-carotenoids. Recombinant EuHCP and EuCTDH had the color of orchids and violets, respectively. The maxima of the absorption spectrum of EuHCP and EuCTDH were 528 and 564 nm, respectively (Fig. 4F), similar to that of CTDH from *Thermosynecoccus elongatus*^11^.

These *Eucbp*s were expressed constitutively in *Euhalothece* cells containing 0% or 3% NaCl (Supplementary Table S2). Interestingly, qRT-PCR analysis revealed that transcript levels of *cruA*, *hcp*, and *ctdh* genes increased upon salt deprivation (Fig. 2), suggesting that carotenoid biosynthesis and CBPs play important roles in *Euhalothece* under conditions of salt deprivation stress.

### Zea and EuCBPs function as antioxidants

**Figure 5:**
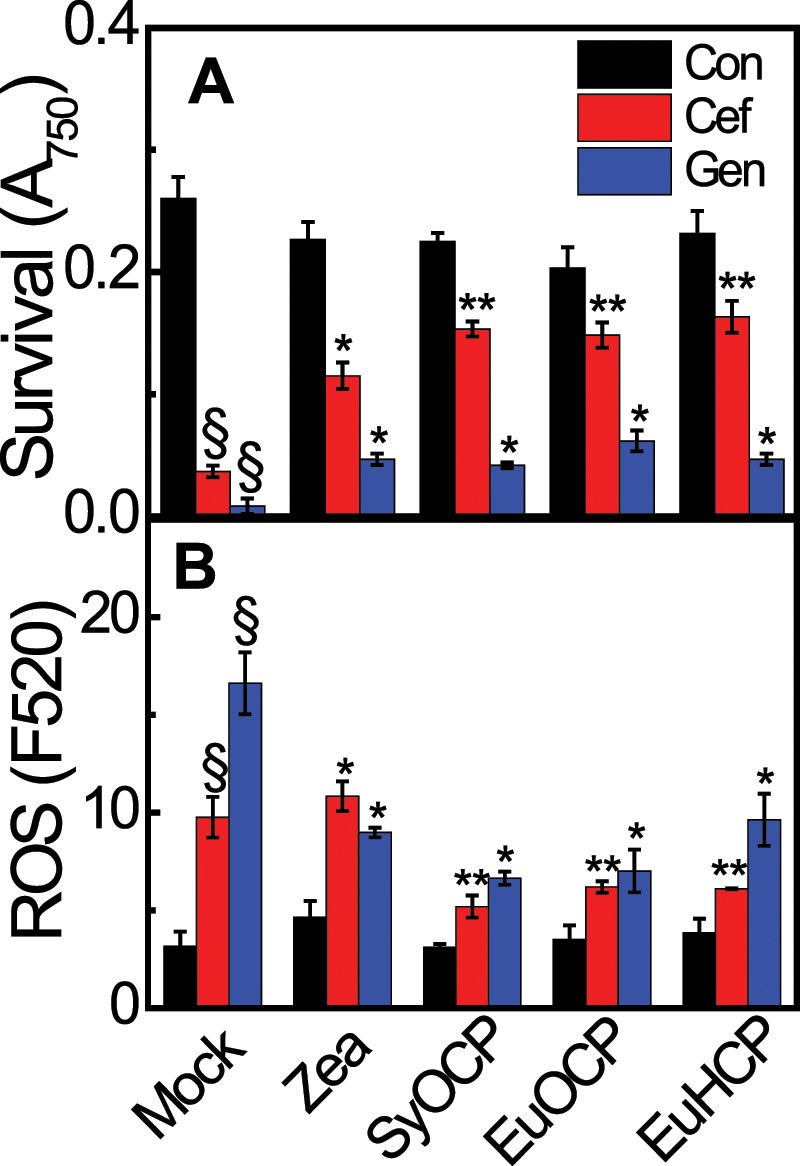
*Euhalothece* carotenoid-binding proteins EuOCP and EuHCP act as ROS scavengers. (**A**,**B**) Survival rate (**A**) and ROS content (**B**) of *E. coli* BL21 cells grown in media containing antibiotics, such as cefatoxime (Cef) and gentamycin (Gen), or DMSO (control; Con). *E. coli* strains produced Zea alone or Zea and CBPs (OCP and HCP) from *Synechocystis* sp. PCC 6803 (SyOCP) and *Euhalothece* Z-M001 (EuOCP and EuHCP). Mock represents *E. coli* cells carrying no plasmid. Data represent mean ± S.E. (*n* = 3–4). ^§^*p < *0.01 for the comparison between cells grown with and without antibiotics (Cef and Gen). *p < 0.05 for the comparison between mock treatment and Zea or Zea + CBP. **p < 0.05 for the comparison between Zea and Zea + CBP.

FRP, which facilitates conversion of red OCP to orange OCP, is absent from the *Euhalothece* genome. Furthermore, Zea-bound OCP is unable to induce fluorescence quenching, although it absorbs blue–green light in crtO-deficient *Synechocystis*^6^. Thus, EuCBPs are most likely inefficient dissipaters of excess light energy. The Zea-binding ability of recombinant EuOCP and EuHCP proteins (Fig. 4A–C) implies that EuCBPs function as ROS scavengers instead of excitation energy quenchers in *Euhalothece* cells. Like other carotenoids, Zea and β-Car are strong ROS scavengers^38^, and OCP and HCP exhibit antioxidant properties^9,39^. Thus, we hypothesized that *E. coli* producing both carotenoids and CBPs would show better survival under ROS stress than wild-type cells. In the present study, ROS production was induced by cefatoxime and gentamycin, which kill bacteria by inducing ROS production^40^. Inhibitory effects of antibiotics on *E. coli* growth were observed; however, the intensity of these effects was low in the presence of Zea alone or Zea-bound OCPs and HCPs. Under antibiotic-induced ROS stress, all three *E. coli* strains containing SyOCP, EuOCP, and EuHCP showed higher survival rates than *E. coli* cells containing only Zea (Fig. 5A). To identify whether the survival rate is related to the ROS scavenging effect of Zea and holo-EuCBPs, we compared the relative ROS content of *E. coli* cells using the fluorescence dye DCFH-DA. Higher ROS content was detected in all *E. coli* cells treated with antibiotics, although *E. coli* cells treated with gentamycin produced more ROS than those treated with cefatoxime. This result explains the lower survival rate of gentamycin-treated *E. coli* cells. After treatment with both antibiotics, ROS content in all three *E. coli* strains containing CBPs was lower than that in *E. coli* cells containing only Zea (Fig. 5B). We did not include CTDH for this *in vitro* assay because CTDH did not bind to Zea (Supplementary Fig. S4)^11^. These results strongly imply that EuCBPs are effective ROS scavengers. Indeed, the absence of ketolase and *cbp* genes is one of the main factors responsible for the sensitivity of *Prochlorococcus* species to high irradiance, and their inability to degrade ROS^41,42^.

### β-Car and Zea content, ROS content, and levels of *crt* and *cbp* gene transcripts at the early phase of salt deprivation

**Figure 6:**
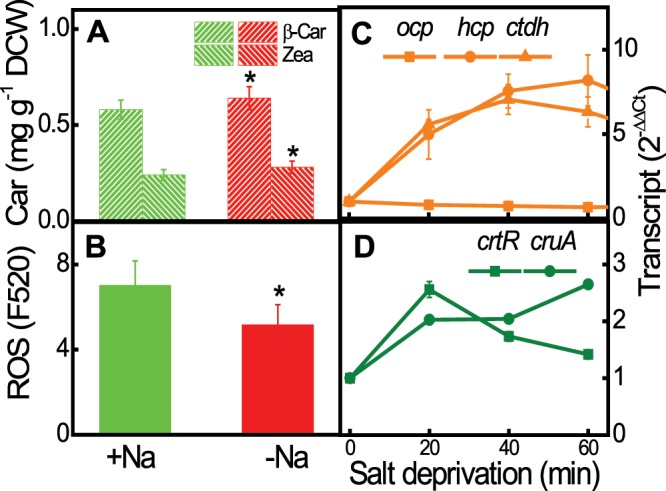
Carotenoid and ROS contents, and transcript levels of genes for carotenogenesis and CBPs in *Euhalothece* Z-M001 during the early phase of salt deficiency. (**A**–**D**) Changes in carotenoid (β-Car and Zea) content (**A**), cellular ROS content (**B**), and expression levels of *ocp, hcp* and *cdth* (**C**), and zeaxanthin (*crtR*), and *β*-carotene (*cruA*) biosynthesis genes (**D**). Cells grown in 3% NaCl (+Na)-containing liquid S-medium were transferred to no-salt (0% NaCl, −Na) medium under growth light conditions for 1 h. Data represent mean ± S.E. (*n* = 3–4). ^§^*p* < 0.01.

The involvement of carotenoids β-Car and Zea, and the corresponding EuCBPs, in ROS scavenging can be inferred from the inverse relationship between carotenoid and ROS content. To verify this inference, *Euhalothece* cells grown in 3% NaCl were transferred to salt-deficient growth medium (0% NaCl) for 1 h. Cells acclimated to salt deficiency for 6 days showed a 4.5-fold increase in total carotenoid content (Fig. 1C); in addition, short-term exposure to salt deprivation increased the content of β-Car and Zea by 1.2–1.3-fold, but decreased that of ROS by 30% (Fig. 6A and B). This inverse relationship implies that carotenoid-dependent ROS scavenging is crucial for responses to salt deprivation. Transcript levels of *hcp* and *ctdh* increased several-fold (Fig. 6C) and remained higher even after acclimation to salt-deficient conditions (Fig. 2); by contrast, those of *ocp* remained unchanged in response to altered salt concentrations (Figs. 2 and 6C). However, expression of *crtR* and *cruA* increased transiently upon salt deprivation (Figs. 2 and 6D). The different responses of carotenoid biosynthesis and CBP genes to salt stress suggests that CBP-dependent ROS scavenging in *Euhalothece* is regulated at both the transcriptional and post-transcriptional level.

In conclusion, among multiple salt acclimation strategies, maintenance of low internal water potential within *Euhalothece* cells is achieved by the MRP system pumping out intracellular Na^+^ ions, a compatible solute glycine betaine and exogenous Na^+^-chelating EPSs. Additionally, *Euhalothece* cells are equipped with β*-*Car- and Zea-dependent ROS scavenging systems against salt stress, whereas Zea either acts freely or is bound to CBPs. Thus, in the absence of *frp*, CBPs seem to act as effective ROS scavengers. The role of EPSs in cell-to-cell aggregation, biofilm formation, biomineralization, and abiotic stress tolerance needs to be explored further in *Euhalothece*.

## Methods

### Strain and cultivation conditions

The natronophilic cyanobacterium *Euhalothece* sp. Z-M001, originally isolated from Lake Magadi (Eastern African Rift Valley, Kenya; 1°52′0″S, 36°16′0″E), was obtained from the Culture Collection of the Department of Biology, Moscow State University^20^. The strain was grown on S-solid media (for 1 L, 1 g KCl, 16.8 g NaHCO_3_, 0.5 g K_2_HPO_4_, 2.5 g NaNO_3_, 1 g K_2_SO_4_, 30 g NaCl, 0.1 g MgSO_4_, 0.04 g CaCl_2_, 0.01 g FeSO_4_, 0.08 g EDTA, 1 mL of trace metal mix stock, and 1% agar [pH 9.5]) or S-liquid media at 28 °C under continuous white light at an intensity of 10 µmol m^−2^s^−1^. To determine the survival rate of *Escherichia coli* cells producing Zea alone or in combination with CBPs such as SyOCP, EuOCP1, and EuHCP, *E. coli* cultures with an optical density of 0.1 at 600 nm (O.D._600_) were treated with cefatoxime (50 μg/ml) or gentamycin (60 μg/ml) and grown in a 6-well plate at 37 °C for 2 h.

### Genomic DNA isolation and purity assessment

Genomic DNA was isolated as described previously^43^. To assess the purity of the extracted genomic DNA, a 16S rRNA gene was amplified by PCR in a 25 μl volume containing 2 mM of each universal 16S rRNA primer (27 F and 1492 R), 200 μM dNTPs, 1 × master mix, and 1 U DNA polymerase (Apex *Taq*; Genesee Scientific Corp.). PCR was performed using the following protocol: initial denaturation at 95 °C for 10 min, followed by 30 cycles of denaturation at 90 °C for 30 s, annealing at 50 °C for 30 s, and extension at 72 °C for 2 min, and a final extension at 72 °C for 5 min. The PCR products were precipitated with ethanol and resuspended in 15 μl nuclease-free water. The 16S rRNA gene amplicon (20–50 ng μl^−1^) was sequenced using commercially available universal primers 27F and 1492R (Supplementary Table S3).

### Whole genome sequencing, assembly, and annotation

Genomic DNA was fragmented and sheared using a g-TUBE (Covaris Inc., Woburn, MA, USA) and then purified using AMPure PB magnetic beads (Beckman Coulter Inc. Bread, CA, USA). A 10 μL library was prepared using the PacBio DNA template Prep Kit 1.0 (for 3–10 kb). SMRTbell templates were annealed using PacBio DNA/Polymerase Binding Kit P6. Libraries were sequenced on PacBio SMRT Cell using the PacBio Sequel (Pacific Biosciences) sequencing platform (Macrogen; Seoul, Korea), yielding 490,426 reads (average read length, 9,180; N50 value, 16,358; total read length, 4,502,006,607 bp). Some reads that were fully contained in other reads provide no extra information with respect to constructing the genome; these were filtered out. After quality control, 81,788 reads were recovered (average length, 5,709; N50 value, 9,479; total read length, 466,969,904 bp). The filtered reads were used to assemble the genome using HGAP4 v4.0, with default options. The genome sequence was circularized using the Circlator algorithm and annotated using the RAST server or the NCBI Prokaryotic Genome Annotation Pipeline (PGAP). The *Circos* program was used to draw the chromosome map. Secondary metabolites were predicted using antiSMASH. CRISPR loci were detected using CRISPRFinder. The genome sequence was deposited in the NCBI BioProject under accession number PRJNA557809.

### RNA isolation and quantitative real-time PCR (qRT-PCR)

Total RNA was isolated from *Euhalothece*, as described previously^44^, with some modifications. The extracted total RNA was treated with DNaseI (Takara) and then cleaned using the NucleoSpin RNA Cleanup Kit (Macherey-Nagel). First-strand cDNA was synthesized using the iScript cDNA Synthesis Kit (Bio-Rad), and qRT-PCR was carried out on the CFX96 Real Time System (Bio-Rad) using SYBR Green Supermix (Bio-Rad) and gene-specific primers (Supplementary Table S3). The 16S rRNA gene was used as the control in each trial of qRT-PCR.

### RNA sequencing and data analysis

Construction of the cDNA libraries, RNA sequencing, and data analysis were carried out as described previously^45^. The Ribo-Zero rRNA Removal Kit (Epicentre, USA) was used to deplete rRNA from 2 μg total RNA. The cDNA libraries were quantified using Bioanalyzer 2100 (Agilent, USA) and sequenced on the Hi-seq 2500 (Illumina, USA) platform to generate 100 bp paired-end reads. The quality of the sequenced reads was evaluated using SolexaQA v1.13, and cleaned reads were extracted by removing low-quality (phred score <20) reads from the dataset^46^. To measure expression of each transcript, cleaned reads were mapped to transcripts using bowtie2 v2.1.0 (mismatch <= 2) and then normalized using the DESeq v1.22.1 library in R^46,47^. Statistical analysis was performed, and genes showing a 2-fold change between compared samples and a false discovery rate *p*-value < 0.01 in a binomial test were identified as differentially expressed. Dendrograms and heatmaps of differentially expressed genes were generated using amap v0.8-14 and gplots v3.0.3 library in R^48,49^.

### Construction of plasmids expressing carotenoid biosynthesis and cbp genes

The PAC-ZEAX plasmid (a.pngt from Prof F. Cunningham)^50^ containing the P15A origin of replication and *crtE*, *crtY*, *crtI*, *crtB*, and *crtZ* genes under the control of the *crtE* promoter from *Erwinia herbicola* Eho10 was used for Zea production in *E. coli*^51^. Genes including *EZR1976* (c*rtR*) from *Euhalothece*, *tpr6673* (*crtW*) from *Tolypothrix* sp. PCC7910, and *slr0088* (*crtO*) from *Synechocystis* were cloned into the PAC-ZEAX plasmid using the *Xho*I and *Kpn*I restriction sites (*ezr1976* and *tpr6673*) or the *Nde*I and *Xho*I restriction sites (*slr0088*). Two genes, *EZR1179* and *EZL1782* (*cruA*), from *Euhalothece* were cloned into pBAD Myc/HisC using *Xho*I and *Hin*dIII sites, whereas genes encoding EuOCP (Ezr1969), EuHCP (Ezr2074), EuCTDH (Ezr2075), and SyOCP (Slr1963) were cloned into pBAD Myc/HisC using *Pst*I and *Eco*RI sites. Primers used for cloning and plasmid construction are listed in Supplementary Table S3.

### Production of carotenoids with or without CBPs in *E. coli*

*E. coli* cells transformed with PAC-ZEAX and PACtrcp-TPR6673 plasmids encoding various carotenoids were grown at 28 °C for 20 h, and Zea or Can production was induced by the addition of 0.2 mM IPTG. Then, Zea- or Can-binding holo-CBPs were produced by the transformation of pBAD-CBP plasmids into Zea- or Can-producing *E. coli* strains. The transformed cells were grown at 37 °C for 20 h. Then, 0.02% arabinose was added to the cultures, and the cells were grown at 28 °C for 20 h. The induced cells were harvested by centrifugation at 3,500 rpm at 4 °C for 10 min, and the cell pellets were stored at −70 °C until needed. LB and RM media were used for BL21 and LMG194 cell cultures, respectively. Chloramphenicol (35 μg/ml) and ampicillin (100 μg/ml) were used for selecting and maintaining the transformed cells.

### Pigment levels

Chlorophyll (Chl) *a*, phycocyanin (PC), and carotenoid (Car) levels in cells grown for 3 days on S-liquid media were estimated using whole-cell spectra^52^ or 100% N,N,-dimethylformamide extracts^53^.

### Carotenoid extraction and HPLC analysis

Carotenoids from lyophilized cyanobacterial cells (15 mg dry cell weight) were extracted using HPLC-grade methanol by sonication for 1 h, followed by centrifugation at 12,000 rpm at 4 °C for 20 min. The supernatants were collected, and the centrifugation was repeated two more times. The supernatants from each centrifugation were pooled, evaporated, concentrated under nitrogen gas, and dissolved in HPLC-grade acetone. The dissolved residues were filtered before HPLC analysis. To extract carotenoids from *E. coli* cell cultures, cold hexane:acetone (1:1, v/v) solution was used^54^. HPLC was performed using an Agilent 1100 HPLC system (Agilent Technologies, Palo Alto, CA, USA) equipped with a PDA detector and YMC carotenoid C_30_ column (5 μm, 250 mm × 4.6 mm; Waters Corp., Milford, MA, USA). The mobile phase consisted of solvent A (92% methanol in 10 mM ammonium acetate) and solvent B (100% methyl tert-butyl ether) at varying ratios^55^. The flow rate and column temperature were maintained at 1 ml/min and 40 °C, respectively. Twenty microliters of each sample was injected into the HPLC machine. To detect carotenoids, the absorbance of samples was measured at 450 nm. Carotenoid species were determined by comparing the peaks of samples with those of standards (CaroteNature Lupsingen, Switzerland). The concentration of each carotenoid was determined as described previously^56^, and all procedures were conducted in darkness.

### Isolation of holo-CBPs

Holo-CBPs were isolated from *E. coli* cells in the dark, as described previously^39^, with some modifications. Frozen pellets of *E. coli* cells were resuspended in the lysis buffer containing 50 mM Tris-HCl (pH 8), 100 mM NaCl, 0.1% IGEPAL, and 10% glycerol, and the mixtures were sonicated for 1–1.5 h. After sonication, the samples were centrifuged at 13,000 rpm for 10 min at 4 °C. The supernatant was transferred to a tube containing Ni-NTA resin, previously resuspended with 300 mM NaCl and 20 mM imidazole at 4 °C for 2 h. After incubation, the Ni-NTA resin was transferred to a column and then washed with 50 mM Tris-HCl buffer (pH 8.0) containing 300 mM NaCl, 0.1% IGEPAL, 10% glycerol, and 20 mM imidazole. CBPs were then eluted using 50 mM Tris-HCl buffer (pH 8.0) containing 300 mM NaCl and 250 mM imidazole. The eluted proteins were stored at −70 °C until needed for further analysis.

### Antibiotic treatment of *E. coli*

*E. coli* BL21 cells carrying the PAC-ZEAX plasmid alone or together with pBAD-CBPs were grown at 37 °C in LB media containing chloramphenicol (35 μg/ml) or both chloramphenicol (35 μg/ml) and ampicillin (200 μg/ml), respectively. After 6 h, the cultures were transferred to 28 °C and then grown for another 12 h. Six hours after the transfer to 28 °C, 0.02% arabinose was added to BL21 cells containing both PAC-ZEAX and pBAD-CBP plasmids, and the cultures were grown for another 6 h. Then, fresh LB media was added to the overnight cultures to dilute them to an O.D._600_ of 0.1. The cultures were grown further to an O.D._600_ of 0.4–0.6; BL21 cells without plasmids were grown at 37 °C, while BL21 cells containing PAC-ZEAX alone or both PAC-ZEAX and pBAD-CBP were grown at 28 °C. The cells were diluted again using 5 mL LB in a 6-well plate (O.D._600_ = 0.1), and cefatoxime (50 μg/ml) or gentamycin (60 μg/ml) was added to the culture. The culture was grown at 37 °C and 130 rpm, and O.D._600_ was measured after 2 h. All cultures were grown in the dark.

### ROS detection

Overnight cultures of *E. coli* were diluted to an O.D._600_ of 0.1 using fresh LB media containing 5 μM 2′,7′-dichlorodihydrofluorescein diacetate (DCFH-DA). *Euhalothece* cells grown in the presence of 3% NaCl or 1.5 h after transfer to salt-deficient S-media were suspended in 1 mL 1X phosphate-buffered saline containing 5 μM DCFH-DA (O.D._580_ = 0.2). Emission spectra were measured using a fluorescence spectrometer (LS-55, Perkin Elmer, Waltham, USA) at an excitation wavelength of 480 nm. Fluorescence emission at 520 nm was used to determine the ROS production.

### Extraction and quantification of exopolysaccharides

Cells grown for 7 days in S-solid medium were inoculated in S-liquid medium containing 3% NaCl or 0% NaCl (O.D_750_ = 0.7). The cultures were grown for 9 days at 28 °C under continuous white light at an intensity of 10 µmol m^−2^s^−1^. Samples (2 ml) were taken from the cultures and centrifuged at 12,000 rpm for 10 min. After washing twice with 1 mL ultra-pure water, the pellets were resuspended in 1 mL ultra-pure water and boiled for 15 min at 100 °C. After cooling and centrifugation at 13,000 rpm for 15 min, supernatants were taken to measure the concentration of total carbohydrate contents of EPSs using the phenol-sulfuric acid method^57^.

### Phylogenetic analysis

Sequences of the 16S rRNA genes of 58 cyanobacteria, whose taxa were already verified^22^, were retrieved from GenBank. The 16S rRNA gene sequences of four additional species (*Euhalothece* Z-M001, *Euhalothece* Z9404, *Microcoleus* IPPAS B-353, and *Nostoc punctiforme* ATCC29133) were confirmed by sequencing and added to the retrieved sequences. Multiple alignments were made using MAFFT ver.7 with the L-INS-i parameter^58^. “The maximum likelihood (ML)” tree was constructed using PhyML and the best-fitting substitution model was selected as GTR + G + I using Akaike information Criterion (AIC)^59–61^.

## Supplementary Information

Supplementary Information.

Supplementary Information 2.
