# Supplementary material for: Genomic Survey of Salt Acclimation-Related Genes in the Halophilic Cyanobacterium Euhalothece sp. Z-M001
Source: Sci Rep. 2020 Jan 20;10:676. doi: 10.1038/s41598-020-57546-1 (PMC6971039; doi:10.1038/s41598-020-57546-1)
Supplement: Supplementary file 1 — Supplementary Information. [file 41598_2020_57546_MOESM1_ESM.docx]

**Supplementary Table S1.** Distribution of genes encoding carotenogenesis and CBPs for orange carotenoid protein (OCP), helical carotenoid protein (HCP), and C-terminal domain homolog (CTDH) from cyanobacterial species with different salt tolerances.

| Organism | Salt tolerance | Carotenoid biosynthesis (NCBI accession no.) | | | Carotenoid binding protein (NCBI accession no.) | | |
| --- | --- | --- | --- | --- | --- | --- | --- |
|  |  | crtR | crtO | crtW | OCP | HCP | CTDH |
| *Acaryochloris marina marina MBIC11017 (T)** | stenohaline (marine) | WP_012164017.1 |  |  |  |  |  |
| *Chamaesiphon minutus* PCC 6605 | stenohaline | WP_015157780.1 |  |  | AFY91616.1 | AFY94389.1 | WP_041549526.1 |
|  |  |  |  |  |  | AFY96489.1 |  |
| *Cyanobacterium aponinum* PCC 10605 (T)* | euryhaline | WP_015218081.1 |  |  |  |  |  |
| *Cyanobacterium stanieri PCC 7202 (T)** | euryhaline | AFZ46762.1 |  |  | AFZ47859.1 |  |  |
| *Cyanobium gracile* PCC 6307 (T)* | stenohaline | AFY29034.1 |  | AFY28028.1 | AFY28029.1 |  |  |
| *Cyanobium* sp. PCC 7001 | euryhaline | WP_006909652.1 |  | WP_006909635.1 | WP_043369152.1 |  | EDY38473.1 |
| *Cyanothece* sp. PCC 7424 | stenohaline | WP_015954388.1 |  |  | ACK72831.1 | ACK69870.1 | WP_015954733.1 |
|  |  |  |  |  | ACK74129.1 | ACK70361.1 |  |
| *Cyanothece* sp. PCC 7425 | stenohaline | WP_012626492.1 | WP_012625910.1 | WP_012630550.1 |  | ACL43998.1 | WP_012627087.1 |
|  |  |  |  |  |  | ACL47114.1 |  |
|  |  |  |  |  |  | ACL42835.1 |  |
| *Cyanothece* sp. PCC 7822 | stenohaline | WP_013325291.1 | ADN15486.1 | ADN16989.1 | ADN16551.1 | ADN12085.1 | WP_013324474.1 |
|  |  |  |  |  |  | ADN12736.1 |  |
|  |  |  |  |  |  | ADN16410.1 |  |
|  |  |  |  |  |  | ADN12638.1 |  |
| *Dactylococcopsis salina* PCC 8305 | stenohaline (halophile) | WP_015229140.1 |  |  | WP_015228119.1 |  |  |
| *Euhalothece* sp. ZM 001 | stenohaline (halophile) | QEI47152.1 |  |  | MK474468 | MK496641 | MK496643 |
| *Euhalothece* sp. Z9404 | stenohaline (halophile) | GenBank |  |  |  | MK496640 | MK496639 |
| *Geminocystis herdmanii* PCC 6308 (T)* | stenohaline | WP_017294837.1 |  |  | WP_017295949.1 |  |  |
| *Gloeobacter violaceus* PCC 7421 (T)* | stenohaline | NP_925569.1 | NP_923340.1 | NP_924674.1 | BAC87991.1 | BAC88200.1 |  |
|  |  |  |  |  | BAC91876.1 | BAC88201.1 |  |
| *Gloeocapsa* sp. PCC 73106 | stenohaline | WP_006528110.1 |  | ELR98653.1 | ELR98495.1 | ELR97197.1 | WP_034937306.1 |
| *Gloeocapsa* sp. PCC 7428 | euryhaline | WP_015187034.1 | WP_015190347.1 | WP_015189329.1 | AFZ30962.1 | AFZ32395.1 | WP_015187560.1 |
|  |  |  |  | WP_015189491.1 |  | AFZ31613.1 |  |
|  |  |  |  |  |  | AFZ29679.1 |  |
|  |  |  |  |  |  | AFZ29683.1 |  |
|  |  |  |  |  |  | AFZ31614.1 |  |
| *Halothece* sp. PCC 7418 | stenohaline (halophile) | WP_015227713.1 |  |  | AFZ44166.1 | AFZ45309.1 | WP_015227182.1 |
| *Microcoleus* sp. B353 | stenohaline (halophile) | GenBank |  |  | MN380640 | MN380641 | MN380642 |
| *Prochlorococcus marinus* AS9601 | stenohaline (marine) | ABM69545.1 |  |  |  |  |  |
| *Prochlorococcus marinus* MIT9202 | stenohaline (marine) | EEE40449.1 |  |  |  |  |  |
| *Prochlorococcus marinus* MIT9211 | stenohaline (marine) | WP_012194815.1 |  |  |  |  |  |
| *Prochlorococcus marinus* MIT9215 | stenohaline (marine) | ABV49875.1 |  |  |  |  |  |
| *Prochlorococcus marinus* MIT9301 | stenohaline (marine) | ABO16881.1 |  |  |  |  |  |
| *Prochlorococcus marinus* MIT9303 | stenohaline (marine) | ABM79163.1 |  |  |  |  |  |
| *Prochlorococcus marinus* MIT9312 | stenohaline (marine) | ABB49299.1 |  |  |  |  |  |
| *Prochlorococcus marinus* MIT9313 | stenohaline (marine) | WP_011131183.1 |  |  |  |  |  |
| *Prochlorococcus marinus* MIT9515 | stenohaline (marine) | ABM71477.1 |  |  |  |  |  |
| *Prochlorococcus marinus* NATL1A | stenohaline (marine) | ABM74879.1 |  |  |  |  |  |
| *Prochlorococcus marinus* NATL2A | stenohaline (marine) | AAZ59091.1 |  |  |  |  |  |
| *Prochlorococcus marinus* subsp. *marinus* CCMP1375 (T)* | stenohaline (marine) | NP_874660.1 |  |  |  |  |  |
| *Prochlorococcus marinus* subsp. *pastoris* CCMP1986 | stenohaline (marine) | CAP16310.1 |  |  |  |  |  |
| *Synechococcus elongatus* PCC 6301 | stenohaline | BAD79857.1 |  |  |  |  |  |
| *Synechococcus elongatus* PCC 7942 | stenohaline | ABB58469.1 |  |  |  |  |  |
| *Synechococcus* sp. PCC 6312 | stenohaline | WP_083853556.1 |  |  | AFY60497.1 |  |  |
| *Synechococcus* sp. PCC 7002 | euryhaline | ACA98919.1 |  | ACB00778.1 | ACB00779.1 |  |  |
| *Synechococcus* sp. PCC 7335 | stenohaline (marine) | WP_006454718.1 | WP_006455611.1 |  | EDX83475.1 | EDX84507.1 |  |
|  |  |  |  |  | EDX83307.1 | EDX87112.1 |  |
|  |  |  |  |  | EDX85092.1 |  |  |
| *Synechococcus* sp. PCC 7336 | stenohaline (marine) | WP_017327296.1 |  |  |  |  |  |
| *Synechococcus* sp. PCC 7502 | stenohaline | WP_015167551.1 |  |  | AFY74032.1 |  |  |
| *Synechocystis* sp. PCC 6803 | euryhaline | WP_020861570.1 | WP_010873789.1 |  | BAA18188.1 |  |  |
| *Synechocystis* sp. PCC 7509 | stenohaline | WP_009633671.1 | WP_009630300.1 |  | WP_028954542.1 | WP_009632401.1 | WP_009630262.1 |
|  |  |  |  |  | WP_009634599.1 | WP_009630016.1 |  |
|  |  |  |  |  | WP_028954515.1 | WP_009631683.1 |  |
| *Chroococcidiopsis thermalis* PCC 7203 | stenohaline | WP_015155217.1 | AFY90837.1 | AFY88302.1 | AFY90822.1 | AFY86582.1 | WP_015155452.1 |
|  |  |  |  |  |  | AFY90873.1 |  |
|  |  |  |  |  |  | AFY85701.1 |  |
|  |  |  |  |  |  | AFY88906.1 |  |
|  |  |  |  |  |  | AFY86581.1 |  |
| *Pleurocapsa* sp. PCC 7319 | stenohaline (marine) | WP_019506156.1 | WP_019505709.1 |  | WP_019505588.1 |  |  |
|  |  |  |  |  | WP_019506750.1 |  |  |
|  |  |  |  |  | WP_019508133.1 |  |  |
| *Pleurocapsa* sp. PCC 7327 | stenohaline | WP_015144704.1 | WP_015143885.1 |  | AFY79747.1 | AFY79754.1 | AFY78316.1 |
|  |  |  |  |  | AFY79757.1 | AFY78317.1 |  |
|  |  |  |  |  |  | AFY77137.1 |  |
| *Stanieria cyanosphaera* PCC 7437 | stenohaline | WP_015192297.1 | WP_015192017.1 |  | AFZ34430.1 | AFZ36878.1 | WP_015194765.1 |
|  |  |  |  |  |  | AFZ36469.1 |  |
| *Xenococcus* sp. PCC 7305 | stenohaline (marine) | WP_006510589.1 |  |  |  | ELS05506.1 | WP_006507407.1 |
| *Coleofasciculus chthonoplastes* PCC 7420 | stenohaline (marine) | WP_006106183.1 |  |  | EDX73443.1 | EDX75031.1 |  |
| *Geitlerinema* sp. PCC 7105 | stenohaline (marine) | WP_017659549.1 |  |  | WP_017660825.1 | WP_017661136.1 | WP_017661135.1 |
|  |  |  |  |  |  | WP_035493682.1 |  |
| *Geitlerinema* sp. PCC 7407 | stenohaline | WP_015171137.1 | WP_015171459.1 |  |  | AFY65112.1 | WP_041268933.1 |
| *Leptolyngbya* sp. PCC 7375 | stenohaline (marine) | WP_006516182.1 |  |  | EKU96221.1 | EKU96226.1 |  |
|  |  |  |  |  | EKU99147.1 | EKV01733.1 |  |
| *Leptolyngbya* sp. PCC 7376 | stenohaline (marine) | WP_015133951.1 |  |  | AFY38316.1 |  |  |
| *Microcoleus* sp. PCC 7113 | stenohaline | WP_015183547.1 | WP_015182905.1 |  | AFZ17625.1 | AFZ21160.1 | WP_015184494.1 |
|  |  |  |  |  |  | AFZ21445.1 |  |
| *Nodosilinea nodulosa* PCC 7104 | euryhaline | WP_026072911.1 |  |  | WP_017298659.1 | WP_017297554.1 | WP_017298117.1 |
|  |  |  |  |  | WP_017301652.1 | WP_017300468.1 |  |
|  |  |  |  |  |  | WP_017301243.1 |  |
| *Oscillatoria* sp. PCC 10802 | stenohaline | WP_017715442.1 |  |  |  |  |  |
| *Pseudanabaena* sp. PCC 7367 | stenohaline (marine) | WP_015164933.1 |  |  | AFY71496.1 |  | WP_041699662.1 |
| *Nostoc punctiforme* PCC 73102 | stenohaline | WP_012410618.1 | ACC79017.1 | ACC83147.1 | ACC83476.1 | ACC84526.1 | WP_012411418.1 |
|  |  |  |  |  | ACC79184.1 | ACC83465.1 |  |
|  |  |  |  |  |  | ACC84206.1 |  |
|  |  |  |  |  |  | ACC83462.1 |  |
| *Rivularia* sp. PCC 7116 | stenohaline (marine) | WP_015121840.1 | WP_015118241.1 | WP_015121931.1 | AFY56555.1 | AFY55621.1 | WP_015117178.1 |
|  |  |  |  |  |  |  |  |
|  |  |  |  |  |  |  |  |

* Type strains

**Supplementary Table S2.** RNA-seq analysis of Euhalothece sp. Z-M001 grown in in S-media with (+Na) or without (-Na) 3% NaCl.

| Classification | Description | Locus | Raw Counts | | Normalized Counts | | Fold change |
| --- | --- | --- | --- | --- | --- | --- | --- |
|  |  |  | -Na | +Na | -Na | +Na | Log2(-Na/+Na) |
| Ion transport |  |  |  |  |  |  |  |
| Na^+^/H^+^ antiporter | Na^+^/H^+^ antiporter (NhaS3) | EZR1753 | - | - | - | - | - |
|  | Na^+^/H^+^ antiporter (NhaS1) | EZR0249 | - | - | - | - | - |
|  | Na^+^/H^+^ antiporter (NhaS4) | EZL0408 | - | - | - | - | - |
|  | Na^+^/H^+^ antiporter (NhaS2) | EZL0594 | - | - | - | - | - |
|  | Na^+^/H^+^ antiporter (NhaS5) | EZR1331 | - | - | - | - | - |
|  | Na^+^/H^+^ antiporter (NhaS6) | EZL1737 | - | - | - | - | - |
| Multisubunit Na^+^/H^+^ antiporter | Multisubunit Na^+^/H^+^ antiporter (MrpB) | EZR1950 | 488 | 504 | 492 | 600 | -0.286 |
|  | Multisubunit Na^+^/H^+^ antiporter (MrpC) | EZR1944 | 140 | 176 | 142 | 210 | -0.564 |
|  | Multisubunit Na^+^/H^+^ antiporter (MrpD) | EZR1945 | 606 | 592 | 610 | 704 | -0.207 |
|  | Multisubunit Na^+^/H^+^ antiporter (MrpE) | EZR1946 | 196 | 154 | 197 | 184 | 0.098 |
|  | Multisubunit Na^+^/H^+^ antiporter (MrpF) | EZR1947 | 94 | 72 | 94 | 86 | 0.128 |
|  | Multisubunit Na^+^/H^+^ antiporter (MrpG) | EZR1948 | 94 | 117 | 94 | 139 | -0.564 |
| K+ transporter | K^+^ transporter (KtrA) | x | - | - | - | - | - |
|  | K^+^ transporter (KtrB | EZL1629 | - | - | - | - | - |
|  | K^+^ transporter (KdpA) | x | - | - | - | - | - |
|  | K^+^ transporter (KdpB) | x | - | - | - | - | - |
|  | K^+^ transporter (KdpC) | x | - | - | - | - | - |
|  | K^+^ transporter (KdpD) | x | - | - | - | - | - |
| K^+^-uptake protein | K^+^ uptake protein (TrkA) | EZL0593 | 1548 | 1996 | 1559 | 2372 | -0.605 |
|  | K^+^ uptake protein (TrkB) | EZL1628 | - | - | - | - | - |
|  |  |  | - | - | - | - | - |
| **Compatible compounds** |  |  |  |  |  |  |  |
| Sucrose | sucrose phosphate synthase (SpsA) | EZL3057 | - | - | - | - | - |
|  | sucrose-phosphate phosphatase (Spp) | EZL0808 | - | - | - | - | - |
|  | sucrose synthase (SuSy) | EZR1815 | 2520 | 1048 | 2537 | 1245 | 1.027 |
| Trehalose | maltooligosyl trehalose trehalohydrolase (TreY, Mth) | EZR0911 | 3878 | 3320 | 3904 | 3946 | -0.015 |
|  | trehalose synthase (TreS) | EZR3241 | 1420 | 1107 | 1430 | 1316 | 0.120 |
| Glucosylglycerol | glucosylglycerol-phosphate synthase (GgpS) | EZR0090 | 21768 | 10545 | 21916 | 12532 | 0.806 |
|  | glucosylglycerol-phosphate phosphatase (GgpP) | EZL3712 | 1312 | 1646 | 1320 | 1956 | -0.567 |
|  | glucosyl 3-phosphoglycerate synthase (GpgS) | x | - | - | - | - | - |
|  | glucosyl 3-phosphoglycerate phosphatase (GpgP) | x | - | - | - | - | - |
| Glycine betaine | glycine-sarcosine-N-methyltransferase (GSMT) | EZR2095 | 11874 | 13978 | 11956 | 16612 | -0.474 |
|  | sarcosine-dimethylglycine-N-methyltransferase (SDMT) | EZR2096 | 11746 | 16684 | 11826 | 19828 | -0.746 |
| proline | glutamate 5-kinase (ProB) | EZL1336 | 818 | 889 | 824 | 1056 | -0.358 |
|  | Gamma-glutamyl phosphate reductase (ProA1) | EZL3204 | 604 | 529 | 608 | 628 | -0.047 |
|  | Gamma-glutamyl phosphate reductase (ProA2) | EZR1928 | 1126 | 705 | 1133 | 838 | 0.435 |
|  | pyrroline-5-carboxylate reductase(ProC) | EZR2844 | 1116 | 882 | 1124 | 1048 | 0.101 |
|  |  |  |  |  |  |  |  |
| **EPS metabolism** |  |  |  |  |  |  |  |
| Polysaccharide Biosynthesis | spore coat polysaccharide biosynthesis protein (SpsA) | EZL0734 | 1861 | 34 | 1874 | 40 | 5.550 |
|  | exopolysaccharide export protein | EZL0725 | 13808 | 442 | 13902 | 526 | 4.724 |
|  | mannosyl transferase | EZL0736 | 2402 | 76 | 2419 | 91 | 4.732 |
|  | haemolysin secretion ATP binding protein | EZL0474 | 1632 | 182 | 1642 | 216 | 2.926 |
|  | galactosyl-1-phosphate transferase | EZL0749 | 311 | 46 | 314 | 55 | 2.513 |
|  | GumB protein | EZL1373 | 6204 | 1604 | 6247 | 1906 | 1.713 |
|  | transcriptional regulator | EZR2682 | 1336 | 578 | 1345 | 687 | 0.969 |
|  | ABC transporter | EZL1258 | 278 | 158 | 280 | 188 | 0.575 |
|  | polysialic acid transport protein(KpsM) | EZL1258 | - | - | - | - | - |
|  | 3-hydroxyisobutyrate dehydrogenase | EZR0664 | 1237 | 636 | 1246 | 756 | 0.721 |
|  | dTDP-glucose 4-6-dehydratase | EZR1418 | 3168 | 1510 | 3190 | 1795 | 0.830 |
|  | glycogen phosphorylase | EZR2172 | 16650 | 9624 | 16764 | 11438 | 0.552 |
|  | unknown protein | EZR1461 | 996 | 600 | 1002 | 713 | 0.491 |
|  | UDP-glucose-4-epimerase | EZL0579 | 2215 | 1054 | 2230 | 1252 | 0.833 |
|  | probable glycosyltransferase | EZL0768 | 794 | 440 | 799 | 524 | 0.609 |
|  | 6-phosphogluconate dehydrogenase | EZL1202 | 3960 | 1418 | 3987 | 1684 | 1.243 |
|  | peptide chain release factor | EZR2860 | 1261 | 797 | 1270 | 947 | 0.423 |
|  | UDP-glucose 4-epimerase | EZL2196 | 7770 | 2800 | 7823 | 3328 | 1.233 |
|  | fructokinase | EZL0450 | 1638 | 1598 | 1648 | 1900 | -0.205 |
|  | ABC transporter | EZL0282 | 3224 | 2460 | 3246 | 2924 | 0.151 |
|  | mannose-6-phosphate isomerase | EZL3312 | 1132 | 548 | 1139 | 652 | 0.805 |
|  | cysteinyl tRNA synthetase | EZR0774 | 2418 | 1578 | 2434 | 1875 | 0.376 |
|  | ABC transporter | EZL2763 | 3812 | 2852 | 3838 | 3390 | 0.179 |
|  | unknown protein | EZL0669 | 1535 | 1144 | 1546 | 1360 | 0.185 |
|  | ABC transporter | EZL0106 | 432 | 205 | 436 | 244 | 0.837 |
|  | 3 isopropylmalate dehydratase | EZL0898 | 1960 | 683 | 1974 | 812 | 1.282 |
|  | cytoplasmic membrane protein for maltose uptake | EZR0783 | 7186 | 4613 | 7236 | 5482 | 0.400 |
|  | urea transport system ATP binding protein | EZR2541 | 1458 | 578 | 1468 | 688 | 1.093 |
|  | imidazoleglycerol-phosphate dehydratase;histidinol-phosphatase | EZL1377 | 476 | 362 | 480 | 430 | 0.159 |
|  | hypothetical protein | EZR3057 | 2308 | 2032 | 2324 | 2416 | -0.056 |
|  | regulatory protein (PchR) | EZL3211 | 152 | 241 | 152 | 286 | -0.912 |
|  | chorismate synthase | EZR3041 | 2946 | 2242 | 2966 | 2664 | 0.155 |
|  | ABC transporter | EZL1259 | 597 | 298 | 601 | 354 | 0.764 |
|  | polysialic acid transport ATP binding protein (KpsT) | EZL1259 | - | - | - | - | - |
|  | ABC transporter | EZL1259 | - | - | - | - | - |
|  | plasmid partitioning protein | EZL2268 | 126 | 100 | 126 | 119 | 0.082 |
|  | acetyltransferase | EZR0108 | 104 | 92 | 104 | 108 | -0.054 |
|  | dTDP-glucose 4,6-dehydratase | EZR1275 | 2467 | 1274 | 2484 | 1513 | 0.715 |
|  | mannosyl transferase | EZL1262 | 878 | 323 | 884 | 384 | 1.203 |
|  | unknown protein | EZL0430 | 112 | 90 | 112 | 108 | 0.052 |
|  | acetate kinase | EZL2648 | 972 | 800 | 978 | 950 | 0.042 |
|  | urea transport system ATP binding protein | EZR2540 | 1448 | 648 | 1458 | 770 | 0.921 |
|  | glycogen operon protein (GlgX) | EZL1004 | 1652 | 1474 | 1663 | 1752 | -0.075 |
|  | ABC transporter | EZR0807 | 747 | 797 | 752 | 947 | -0.333 |
|  | GDP-mannose pyrophosphorylase | EZL2289 | 876 | 553 | 882 | 657 | 0.425 |
|  | HlyB family | EZL1255 | 6906 | 3388 | 6954 | 4026 | 0.788 |
|  | phosphate transport ATP-binding protein (PstB) | EZL3176 | 944 | 535 | 951 | 636 | 0.580 |
|  | melibiose carrier protein | EZR1677 | 1872 | 1222 | 1884 | 1453 | 0.375 |
|  | ATP-binding subunit of an ABC-type osmolyte transporter | EZL2531 | 658 | 776 | 662 | 922 | -0.478 |
|  | probable plasmid partitioning protein, ParA family | EZR3322 | 507 | 872 | 510 | 1036 | -1.022 |
|  | ParA family chromosome partitioning protein | EZR3322 | - | - | - | - | - |
|  | glycogen synthase | EZL3158 | 1775 | 1793 | 1788 | 2131 | -0.253 |
|  | glucose 6-phosphate dehydrogenase | EZR2036 | 6018 | 3428 | 6058 | 4074 | 0.572 |
|  | ABC transporter | EZR1836 | 6561 | 5994 | 6606 | 7124 | -0.109 |
|  | phosphotransacetylase | EZR1680 | 1922 | 1874 | 1936 | 2226 | -0.201 |
|  | NarL subfamily | EZL1241 | 4559 | 3538 | 4590 | 4205 | 0.126 |
|  | serine acetyltransferase | EZL22730 | 2308 | 1882 | 2324 | 2236 | 0.056 |
|  | phosphomannose isomerase | EZR3079 | 703 | 827 | 708 | 983 | -0.473 |
|  | ABC transporter | EZR2080 | 89 | 68 | 90 | 80 | 0.170 |
|  | glucose 1 phosphate thymidylyltransferase | EZR1272 | 2436 | 2611 | 2452 | 3103 | -0.340 |
|  | cytochrome c oxidase folding protein | EZL0805 | 894 | 424 | 900 | 504 | 0.837 |
|  | ABC transporter | EZL0926 | 1746 | 1124 | 1758 | 1335 | 0.397 |
|  | peptide chain release factor | EZR2609 | 5165 | 3230 | 5200 | 3838 | 0.438 |
|  | ABC transporter | EZL3214 | 1363 | 686 | 1372 | 816 | 0.750 |
|  | ribose 5-phosphate isomerase | EZR1937 | 1130 | 581 | 1138 | 690 | 0.722 |
|  | ABC transporter | EZR2421 | 621 | 322 | 625 | 382 | 0.710 |
|  | hybrid sensory kinase | EZR1462 | 1151 | 1138 | 1158 | 1353 | -0.225 |
|  | ABC transporter | EZR0112 | 798 | 1096 | 803 | 1302 | -0.697 |
|  | L argininosuccinate lyase | EZL2925 | 642 | 612 | 647 | 727 | -0.168 |
|  | similar to exopolysaccharide export protein | EZL2830 | 1440 | 1319 | 1450 | 1568 | -0.113 |
|  | 5 oxo 1,2,5 tricarboxilic 3 penten aciddecarboxilase/isomer | EZR0336 | 1425 | 1086 | 1434 | 1290 | 0.153 |
|  | hypothetical protein | EZL1263 | 1034 | 818 | 1041 | 972 | 0.099 |
|  | mannose 1 phosphate guanyltransferase | EZL2337 | 19824 | 8923 | 19960 | 10605 | 0.912 |
|  | unknown protein | EZL2337 | - | - | - | - | - |
|  | spore coat polysaccharide biosynthesis protein (SpsC) | EZL1102 | 1178 | 980 | 1186 | 1165 | 0.026 |
|  | aldehyde reductase | EZR0878 | 3588 | 3912 | 3613 | 4650 | -0.364 |
|  | phosphofructokinase | EZL1509 | 396 | 578 | 399 | 688 | -0.786 |
|  | ABC transporter | EZL3221 | 2846 | 2042 | 2866 | 2426 | 0.240 |
|  | 1,4-alpha-glucan branching enzyme | EZL1013 | 1596 | 1230 | 1608 | 1462 | 0.137 |
|  | UDP 3-O-(3-hydroxymyristoyl)glucosaminen acyltransferase | EZR1314 | 890 | 700 | 896 | 832 | 0.107 |
|  | ferredoxin | EZR2833 | 873 | 540 | 879 | 642 | 0.453 |
|  | phosphoglycerate dehydrogenase | EZL0216 | 5860 | 4788 | 5900 | 5690 | 0.052 |
|  | alanyl tRNA synthetase | EZR0455 | 2442 | 2146 | 2459 | 2550 | -0.052 |
|  | ABC transporter | EZL2840 | 440 | 432 | 444 | 514 | -0.211 |
|  | transketolase | EZR2972 | 43108 | 55821 | 43404 | 66344 | -0.612 |
|  | hybrid sensory kinase | EZR0612 | 2454 | 2231 | 2470 | 2652 | -0.103 |
|  | unknown protein | EZR1457 | 2380 | 2052 | 2396 | 2438 | -0.025 |
|  | mannose 1 phosphate guanyltransferase | EZL2533 | 3888 | 2758 | 3914 | 3277 | 0.256 |
|  | nitrogen regulatory protein P II | EZL0332 | 6620 | 7442 | 6665 | 8844 | -0.408 |
|  | dTDP-4-dehydrorhamnose 3,5-epimerase | EZR1273 | 698 | 458 | 702 | 544 | 0.368 |
|  | thioredoxin | EZL1943 | 11743 | 13162 | 11822 | 15642 | -0.404 |
|  | hybrid sensory kinase | EZR2455 | 900 | 1115 | 906 | 1325 | -0.548 |
|  | phosphoglucomutase | EZR1120 | 2272 | 2526 | 2287 | 3003 | -0.393 |
|  | RfbJ protein | EZR1211 | 334 | 339 | 337 | 403 | -0.258 |
|  | thioredoxin M | EZR1622 | 430 | 156 | 432 | 186 | 1.216 |
|  | methyl-accepting chemotaxis protein II | EZL0257 | 4976 | 5274 | 5010 | 6268 | -0.323 |
|  | 2,3-bisphosphoglycerate-independentphosphoglycerate mutase | EZR2610 | 4214 | 3773 | 4244 | 4484 | -0.079 |
|  | nitrate transport protein | EZL0277 | 167 | 107 | 168 | 127 | 0.404 |
|  | sulfate transport system permease protein | EZR1579 | 752 | 734 | 758 | 872 | -0.202 |
|  | ABC transporter subunit | EZL1030 | 3507 | 1746 | 3531 | 2076 | 0.766 |
|  | citrate synthase | EZL2128 | 962 | 766 | 968 | 910 | 0.089 |
|  | NarL subfamily | EZR2999 | 2331 | 1188 | 2347 | 1412 | 0.733 |
|  | dTDP-6-deoxy-L-mannose-dehydrogenase | EZR1274 | 2647 | 3855 | 2664 | 4582 | -0.782 |
|  | thioredoxin | EZL0441 | 662 | 552 | 666 | 656 | 0.022 |
|  | sugar fermentation stimulation protein | EZR1963 | 1031 | 886 | 1038 | 1054 | -0.022 |
|  | acetyl-coenzyme A synthetase | EZR2871 | 11927 | 16682 | 12008 | 19826 | -0.723 |
|  | NarL subfamily | EZR1962 | 3636 | 5908 | 3660 | 7022 | -0.940 |
|  | glycolate oxidase subunit (GlcD) | EZR0212 | 2356 | 2082 | 2372 | 2474 | -0.061 |
|  | Ferredoxin | EZL1880 | 17111 | 26591 | 17226 | 31602 | -0.875 |
|  | ATP-binding protein of molybdate ABC transporter | EZR1035 | 244 | 384 | 246 | 456 | -0.890 |
|  | GDP-D-mannose dehydratase | EZL1751 | 3451 | 3285 | 3474 | 3904 | -0.168 |
|  | GDP-fucose synthetase | EZL1752 | 1016 | 1274 | 1024 | 1514 | -0.564 |
|  | ferredoxin | EZR1039 | 236 | 200 | 237 | 238 | -0.006 |
|  | glucosyltransferase | EZR2732 | 251 | 4287 | 253 | 5095 | -4.332 |
|  |  |  |  |  |  |  |  |
| **Carotenoids and CBPs** |  |  |  |  |  |  |  |
| Carotenoid Biosynthesis | GGPP synthase (CrtE) | EZR2484 | 692 | 500 | 696 | 594 | 0.229 |
|  | phytoene synthase (CrtB) | EZL1546 | 1073 | 1504 | 1080 | 1787 | -0.727 |
|  | phytoene desaturase(CrtP) | EZL1545 | 2796 | 3486 | 2816 | 4143 | -0.557 |
|  | carotene 7,8-desaturase (CrtQ) | EZL1380 | 1292 | 1079 | 1301 | 1282 | 0.021 |
|  | carotenoid isomerase) (CrtH) | EZR0040 | 1035 | 804 | 1042 | 955 | 0.126 |
|  | lycopene cyclase (CruA) | EZL1782 | 1370 | 1044 | 1380 | 1240 | 0.154 |
|  | lycopene cyclase (CruP) | EZR1179 | 1382 | 2382 | 1392 | 2736 | -0.975 |
|  | C1' hydroxylase (CruF) | EZL1914 | 2363 | 1625 | 2379 | 1931 | 0.301 |
|  | C2'-O-glycosidase (CruG) | EZL1915 | 2476 | 1427 | 2493 | 1696 | 0.556 |
|  | beta-carotene hydroxylase(CrtR) | EZR1976 | 954 | 928 | 960 | 1102 | -0.199 |
|  | carotenoid oxygenase | EZL0469 | 5702 | 8329 | 5742 | 9899 | -0.786 |
|  | carotenoid oxygenase | EZL3063 | 814 | 324 | 820 | 386 | 1.087 |
| Carotenoid binding protein | Orange carotenoid protein (OCP) | EZR1969 | 20432 | 21057 | 20572 | 25026 | -0.283 |
|  | Helical carotenoid protein (HCP) | EZR2074 | 3048 | 2506 | 3068 | 2979 | 0.042 |
|  | C-terminal domain homolog (CTDH) | EZR2075 | 366 | 295 | 369 | 350 | 0.076 |
|  |  |  |  |  |  |  |  |
| **Histidine kinase** |  |  |  |  |  |  |  |
|  | Multi-sensor hybrid histidine kinase | EZL0264 | 1397 | 848 | 1406 | 1008 | 0.480 |
|  | PAS/PAC sensor signal transduction histidine kinase | EZL0642 | 240 | 288 | 241 | 343 | -0.509 |
|  | Multi-sensor hybrid multi-kinase | EZL0644 | 2032 | 2100 | 2046 | 2496 | -0.287 |
|  | Histidine kinase (Precursor) | EZL0966 | 122 | 178 | 122 | 212 | -0.797 |
|  | Periplasmic sensor signal transduction histidine kinase (Precursor) | EZL0967 | 200 | 180 | 202 | 214 | -0.083 |
|  | Sensory transduction histidine kinase | EZL1011 | 1728 | 1102 | 1740 | 1310 | 0.410 |
|  | CheA signal transduction histidine kinase | EZL1843 | 6927 | 7600 | 6974 | 9032 | -0.373 |
|  | Putative signal transduction histidine kinase | EZL1858 | 1466 | 2800 | 1476 | 3328 | -1.173 |
|  | Histidine kinase | EZL2094 | 804 | 869 | 810 | 1033 | -0.351 |
|  | Two-component hybrid sensor and regulator | EZL2163 | 706 | 681 | 710 | 810 | -0.190 |
|  | CBS sensor hybrid histidine kinase | EZL2413 | 952 | 786 | 958 | 934 | 0.037 |
|  | Integral membrane sensor signal transduction histidine kinase (Precursor) | EZL2451 | 708 | 452 | 712 | 536 | 0.410 |
|  | Two-component sensor histidine kinase | EZL2773 | 1207 | 1506 | 1215 | 1790 | -0.559 |
|  | Putative CheA signal transduction histidine kinase | EZL2953 | 3344 | 4030 | 3367 | 4790 | -0.509 |
|  | Response regulator receiver sensor signal transduction histidine kinase | EZL3071 | 782 | 211 | 787 | 251 | 1.649 |
|  | Response regulator receiver sensor signal transduction histidine kinase | EZL3152 | 226 | 182 | 228 | 217 | 0.071 |
|  | GAF sensor signal transduction histidine kinase | EZR0141 | 745 | 900 | 750 | 1069 | -0.511 |
|  | Two-component sensor histidine kinase | EZR0330 | 500 | 549 | 504 | 652 | -0.371 |
|  | CheA signal transduction histidine kinase | EZR0416 | 640 | 756 | 644 | 898 | -0.480 |
|  | CheA-like two-component hybrid sensor and regulator | EZR0417 | 142 | 102 | 143 | 121 | 0.241 |
|  | Response regulator receiver sensor signal transduction histidine kinase | EZR0611 | 469 | 603 | 472 | 716 | -0.601 |
|  | Signal transduction histidine kinase | EZR0612 | 2454 | 2231 | 2470 | 2652 | -0.103 |
|  | Multi-sensor signal transduction histidine kinase | EZR0922 | 6420 | 6102 | 6464 | 7253 | -0.166 |
|  | Putative CheA signal transduction histidine kinases | EZR1145 | 568 | 462 | 572 | 548 | 0.062 |
|  | Two-component sensor histidine kinase | EZR1148 | 1939 | 944 | 1952 | 1122 | 0.799 |
|  | Adaptive-response sensory-kinase sasA | EZR1237 | 1048 | 1103 | 1056 | 1310 | -0.311 |
|  | Two-component sensor histidine kinase | EZR1462 | 1151 | 1138 | 1158 | 1353 | -0.225 |
|  | Histidine triad family protein | EZR2313 | 456 | 274 | 458 | 325 | 0.495 |
|  | Two-component hybrid sensor and regulator | EZR2455 | 900 | 1115 | 906 | 1325 | -0.548 |
|  | SipA; possible regulator of histidine kinase NblS | EZR2645 | 952 | 579 | 958 | 688 | 0.478 |
|  | Integral membrane sensor signal transduction histidine kinase | EZR2811 | 1158 | 2788 | 1166 | 3313 | -1.507 |
|  | Putative CheA signal transduction histidine kinase | EZR2817 | 11723 | 9684 | 11804 | 11510 | 0.036 |
|  | Two-component hybrid sensor and regulator | EZR3515 | 488 | 220 | 492 | 262 | 0.909 |

x genes not found; -, not mapped

**Supplementary Table S3.** *Escherichia coli* strains, plasmids, and primers used in this study

| **Strain, plasmid, or primer** | **Deviation and/or relevant characteristics** | **Reference or source** |
| --- | --- | --- |
| ***E. coli* strains** |  |  |
| BL21 | F－, ompT, hsdS B (rB－mB－), gal, dcm | Takara |
| LMG194 | St^r^, Tet^r^, F- ∆lacX74 gal E thi rpsL ∆phoA (Pvu II) ∆ara714 leu::Tn10 | Guzman et al. (1995), Invitrogen |
|  |  |  |
| **Plasmids** |  |  |
| pAC-ZEAX | Chl^r^, Deletion of a 1.1 kb *Sal*I–*Sal*I fragment containing *Erwinia herbicola* Eho10 gene, *crtX*, from PAC-EHER. | Sun et al. (1996) |
| pAC-BETA | Chl^r^, Deletion of 0.8 and 1.1 kb *Bam*HI–*Bam*HI fragments from pAC-ZEAX. | Cunningham et al. (1996) |
| pTrcHis | Ap^r^, cloning vector | This study |
| pACtrcp | Chl^r^, pTrcHIS, in which the GM cassette was ligated to the promoter by blunt ligation and inserted into pAC-BETA. | This study |
| pACtrcp-ezr1976 | Chl^r^, PCR fragment containing *ezr1976* (*crtR*) cloned into PACtrcp using *Xho*I and *Kpn*I restriction sites. | This study |
| pACtrcp-tpr6673 | Chl^r^, PCR fragment containing *tpr6673* (*crtW*) cloned into PAC trcp using *Xho*I and *Kpn*I sites. | This study |
| pACtrcp-slr0088 | Chl^r^, PCR fragment containing *Slr0088* (*crtO*) cloned into PACtrcp using *Nde*I and *Xho*I sites. | This study |
| pACtrcp-LYC | Chl^r^, 5.0 kb *Apa*LI–*Sal*I fragment containing *crtY*, *crtI*, and *crtB* was deleted from pACtrcp and replaced by a 2.4 kb fragment containing *crtI* and *crtB* amplified from pACtrcp. | Cunningham et al. (1994) |
| pBAD Myc/HisC | Ap^r^, cloning vector | Guzman et al. (1995) |
| pBAD-CBP | Ap^r^, PCR fragment containing *Slr1963*, *EZR1969*, *EZR2075*, or *EZR2074* cloned into pBAD Myc/HisC using *Pst*I and *Eco*RI sites. Among the four genes inserted into the vector, three (*ezr1969*, *ezr2075*, and *ezr2074*) contained 6X-His tag at the C-terminal end, whereas *slr1963* carried the 6X-His tag at its N-terminal end. | Muzzopappa et al. (2017), López-Igual et al. (2016), De Carbon et al. (2015) |
| pBAD-ezr1179 | Ap^r^, PCR fragment containing ezr*1179* cloned into pBAD Myc/HisC using *Xho*I and *Hin*dIII sites. | This study |
| pBAD-ezl1782 | Ap^r^, PCR fragment containing ezl*1782* cloned into pBAD Myc/HisC using *Xho*I and *Hin*dIII sites. | This study |
|  |  |  |
| **Primers for qRT-PCR** | **Sequences (5'→3')** |  |
| 16S rRNAF | CTA ATC CCC AAT GTG CCG AA | This study |
| 16S rRNAR | TGC TCA TCC TCT CAG ACC AG | This study |
| EZL1782F | TTG ATA GTG TCT GCC CAA CG | This study |
| EZL1782R | GAA ATA ACT CCC AAA TCA GTT | This study |
| EZR1976F | ACT CGG GTT CAT TTA CAG C | This study |
| EZR1976R | CGG GAA TCA GAA ACA GTG GC | This study |
| EZR1969F | TTT GGT TTG CTT ACA AGG AG | This study |
| EZR1969F | CTT GTA AAA CAG AAG AAG CAT T | This study |
| EZR2074F | GGC TCA GTG GAT GGA AGA TG | This study |
| EZR2074R | GAC GGA GAA TGG TGA TTT GC | This study |
| EZR2075F | TCC ACT GCT TCT CTT ACC CA | This study |
| EZR2075R | ATC GCC AAT AAC AGG TTC TTC AA | This study |
| EZR1815F | ATT GTC CTT GTC TCC GTT CAT G | This study |
| EZR1815 R | ATT GTC CTT GTC TCC GTT CAT | This study |
| EZR3241 F | GAT TAT TTG GCG TGG GAG GTG | This study |
| EZR3241 R | CAG GAA AGA GAT AAC GGG TGG TG | This study |
| EZR0090 F | TTC CAT CAC ACC CCA TTC CCC TC | This study |
| EZR0090 F | GTC TTG TTC CAC TAC TTC TAC CTT AC | This study |
| EZR2095 F | AGT GTT GAT GGT AGC GTT GAG AT | This study |
| EZR2095 R | CAG AGA AAA GAT GAG TAA AGG AGT T | This study |
|  |  |  |
| **Primers for cloning** | **Sequences (5'→3')** |  |
| SyOCPpBADpstIF | TTT CTG CAG AAA TGC CAT TCA CCA TTG ACT CTG | De Carbon et al. (2015) |
| SyOCPpBADEcoRIR | AAA GAA TTC TTA GTG GTG GTG GTG GTG GTG GCG AGC AAA GTT GAG TAA TTC | De Carbon et al. (2015) |
| EuOCPpBADPstIF | TTT CTG CAG AAA TGC CTT TTA GTA TCG AGT C | This study |
| EuOCPpBADEcoRIR | AAA GAA TTC TTA GTG GTG GTG GTG GTG GTG GCG AGA AAG ATT CAT TAA CTC T | This study |
| EuHCPpBADpstIF | AAA CTG CAG AAA TGG TTG CTA CTA CCA ATA C | This study |
| EuHCPpBADEcoRIR | AAA GAA TTC TTA GTG GTG GTG GTG GTG GTG AGC AAA CGC ATC AAC GC | This study |
| EuCTDHpBADpstIF | AAA CTG CAG AAA TGT CCA CTG CTT CTC TTA | This study |
| EuCTDHpBADEcoRIR | AAA GAA TTC TTA GTG GTG GTG GTG GTG GTG ACG TTG AAT TTG GGC AAG | This study |
| ezr1179pBADXhoIF | TAC TCG AGA ATG AAG CCA ACC GAA CAA ATC | This study |
| ezr1179pBADHindIIIR | TT TTT AAG CTT C ATC CTG TCC TGA TCC ATA TTG | This study |
| ezl1782pBADXhoIF | TTA CTC GAG AAT GGA AGA AAT TCT CTA CAT TGA | This study |
| ezl1782pBADHindIIIR | TT TTT AAG CTT C ATT TTT GCT TCT AAC CTT CCC | This study |
| ezr1976XhoIF | AAC TCG AGA ATG GTG CAG ATG CAA CAG | This study |
| ezr1976KpnIR | TTG GTA CCT GA T TTA TTT TCT TTT TTA TGA AAG CG | This study |
| slr0088NdeIF | TTT TCA TAT GAT GAT CAC CAC CGA TGT TG | This study |
| slr0088XhoIR | TAT CTC GAG TTA CCA AAA ACG ACG TTG TTG | This study |
| ToCrtW2-XhoIF | CC CTCGAG ATGATTCAGTTAGAACAAGC | This study |
| ToCrtW2-KpnIR | GG GGTACC CAA TTT ATT TTT AGA TAT TG | This study |
| pACBETAAPalIF | AAA GTG CAC CAT GAA A AA A AC CGT TGT GAT TGG C | Cunningham et al. (1994) |
| pACBETASalIR | AAA GTC GAC GAT CCT GCG TGA ACG TCA TG | Cunningham et al. (1994) |
| TrcPFHindIII | TTT TAA GCT TTT CTG AAA TGA GCT GTT GAC | This study |
| TrcPR2HindIII | TTT TAA GCT TTT AGG TAC CAC CGC TCG AGC CAG CCA TAT GGG TTT ATT CCT CCT TAT TTA ATC G | This study |
