# Supplementary material for: Genomic Survey of Salt Acclimation-Related Genes in the Halophilic Cyanobacterium Euhalothece sp. Z-M001
Source: Sci Rep. 2020 Jan 20;10:676. doi: 10.1038/s41598-020-57546-1 (PMC6971039; doi:10.1038/s41598-020-57546-1)
Supplement: Supplementary file 2 — Supplementary Information 2. [file 41598_2020_57546_MOESM2_ESM.pdf]

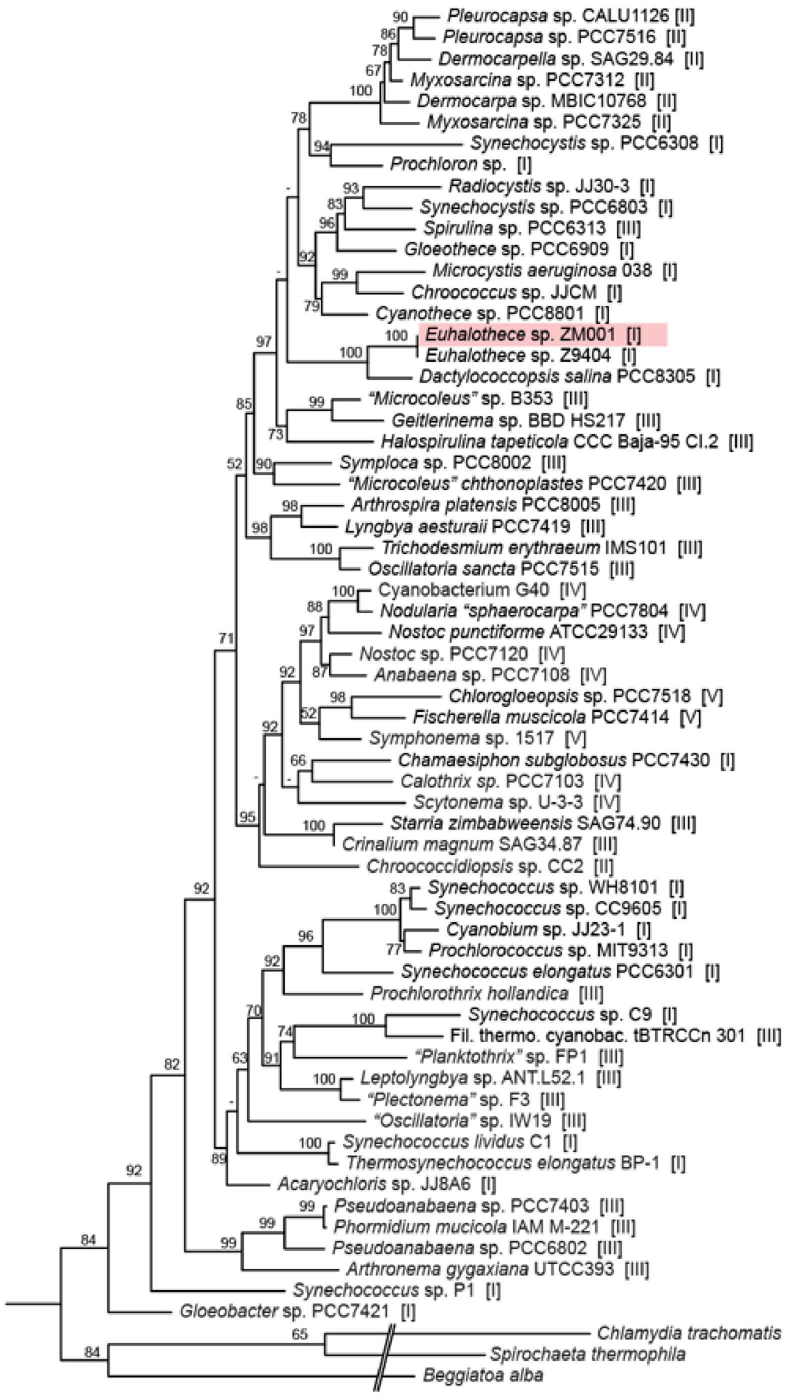


**Supplementary Figure S1. Phylogenetic analysis of 65 cyanobacterial species including *Euhalothece* sp. Z-M001.** Sequences of 16S rRNA genes from euhalophilic cyanobacteria *Euhalothece* Z-M001, *Euhalothece* sp. Z9404, and *Microcoleus* IPPAS B-353 were added to those of 58 cyanobacteria used previously for phylogenetic construction[1]. *Chlamydia trachomatis*, *Spirochaeta thermophila*, and *Beggiato alba* were used as outgroups.

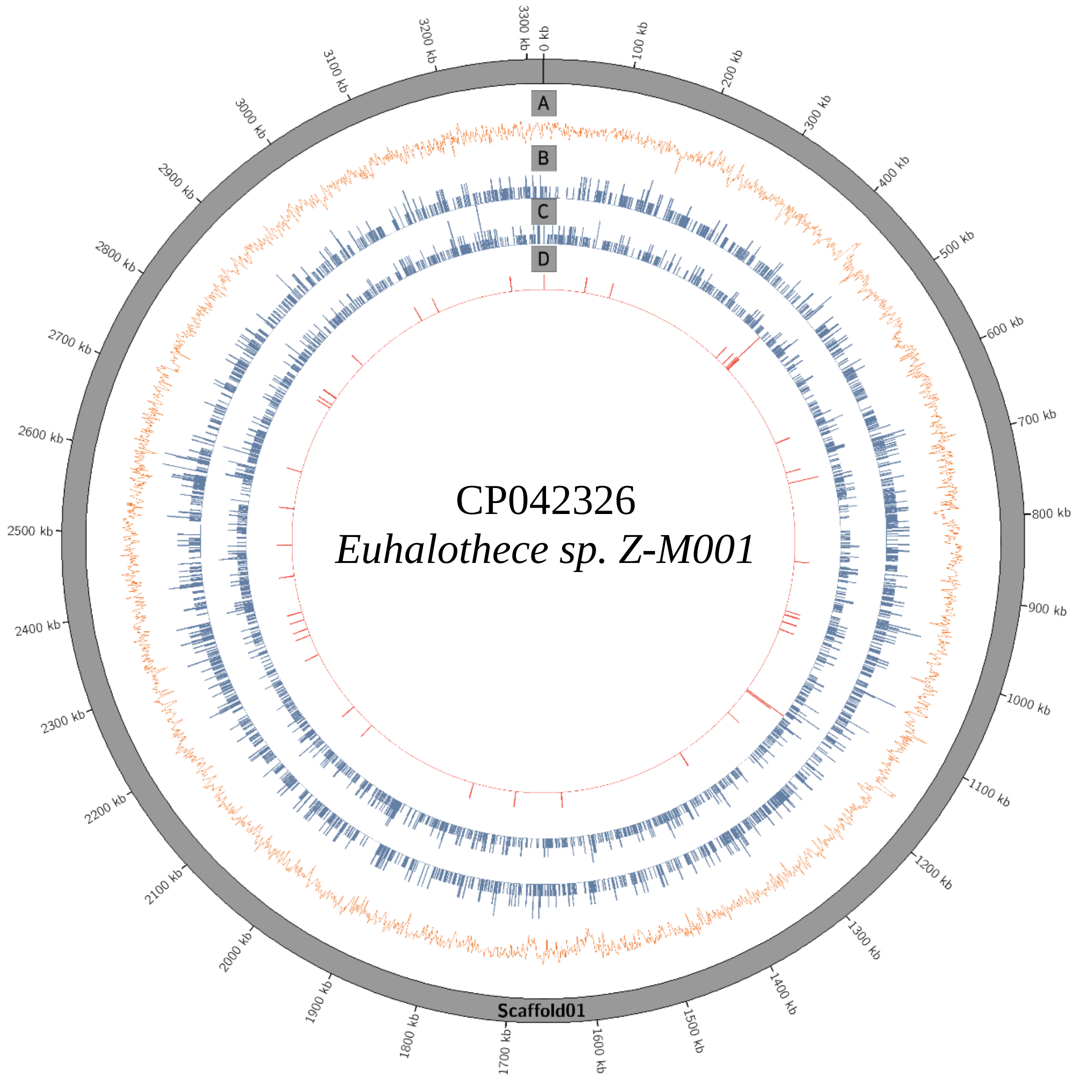


**Supplementary Figure S2. Map of the circular chromosome of *Euhalothece* Z-M001.**

Marked characteristics are shown from outside to the center: GC content (A), CDS on reverse strand (B), CDS on forward strand (C), and tRNA and rRNA (D). The circular genome map was drawn using *with* the *Circos* program v0.63-4 (http://circos.ca/).

A

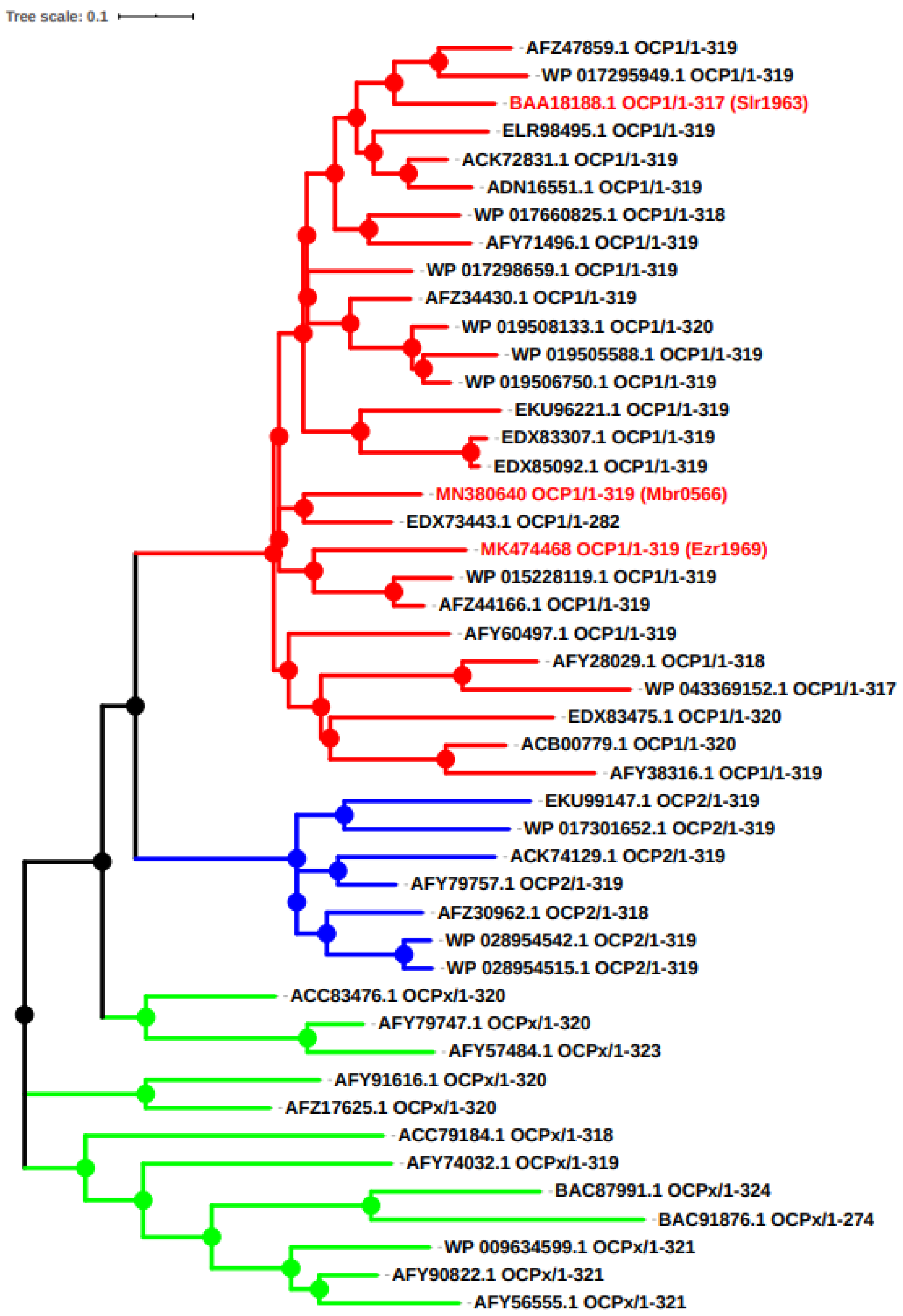
Tree scale: 0.1
AFZ47859.1 OCP1/1-319
WP 017295949.1 OCP1/1-319
BAA18188.1 OCP1/1-317 (Slr1963)
ELR98495.1 OCP1/1-319
ACK72831.1 OCP1/1-319
ADN16551.1 OCP1/1-319
WP 017660825.1 OCP1/1-318
AFY71496.1 OCP1/1-319
WP 017298659.1 OCP1/1-319
AFZ34430.1 OCP1/1-319
WP 019508133.1 OCP1/1-320
WP 019505588.1 OCP1/1-319
WP 019506750.1 OCP1/1-319
EKU96221.1 OCP1/1-319
EDX83307.1 OCP1/1-319
EDX85092.1 OCP1/1-319
MN380640 OCP1/1-319 (Mbr0566)
EDX73443.1 OCP1/1-282
MK474468 OCP1/1-319 (Ezr1969)
WP 015228119.1 OCP1/1-319
AFZ44166.1 OCP1/1-319
AFY60497.1 OCP1/1-319
AFY28029.1 OCP1/1-318
WP 043369152.1 OCP1/1-317
EDX83475.1 OCP1/1-320
ACB00779.1 OCP1/1-320
AFY38316.1 OCP1/1-319
EKU99147.1 OCP2/1-319
WP 017301652.1 OCP2/1-319
ACK74129.1 OCP2/1-319
AFY79757.1 OCP2/1-319
AFZ30962.1 OCP2/1-318
WP 028954542.1 OCP2/1-319
WP 028954515.1 OCP2/1-319
ACC83476.1 OCPx/1-320
AFY79747.1 OCPx/1-320
AFY57484.1 OCPx/1-323
AFY91616.1 OCPx/1-320
AFZ17625.1 OCPx/1-320
ACC79184.1 OCPx/1-318
AFY74032.1 OCPx/1-319
BAC87991.1 OCPx/1-324
BAC91876.1 OCPx/1-274
WP 009634599.1 OCPx/1-321
AFY90822.1 OCPx/1-321
AFY56555.1 OCPx/1-321

B

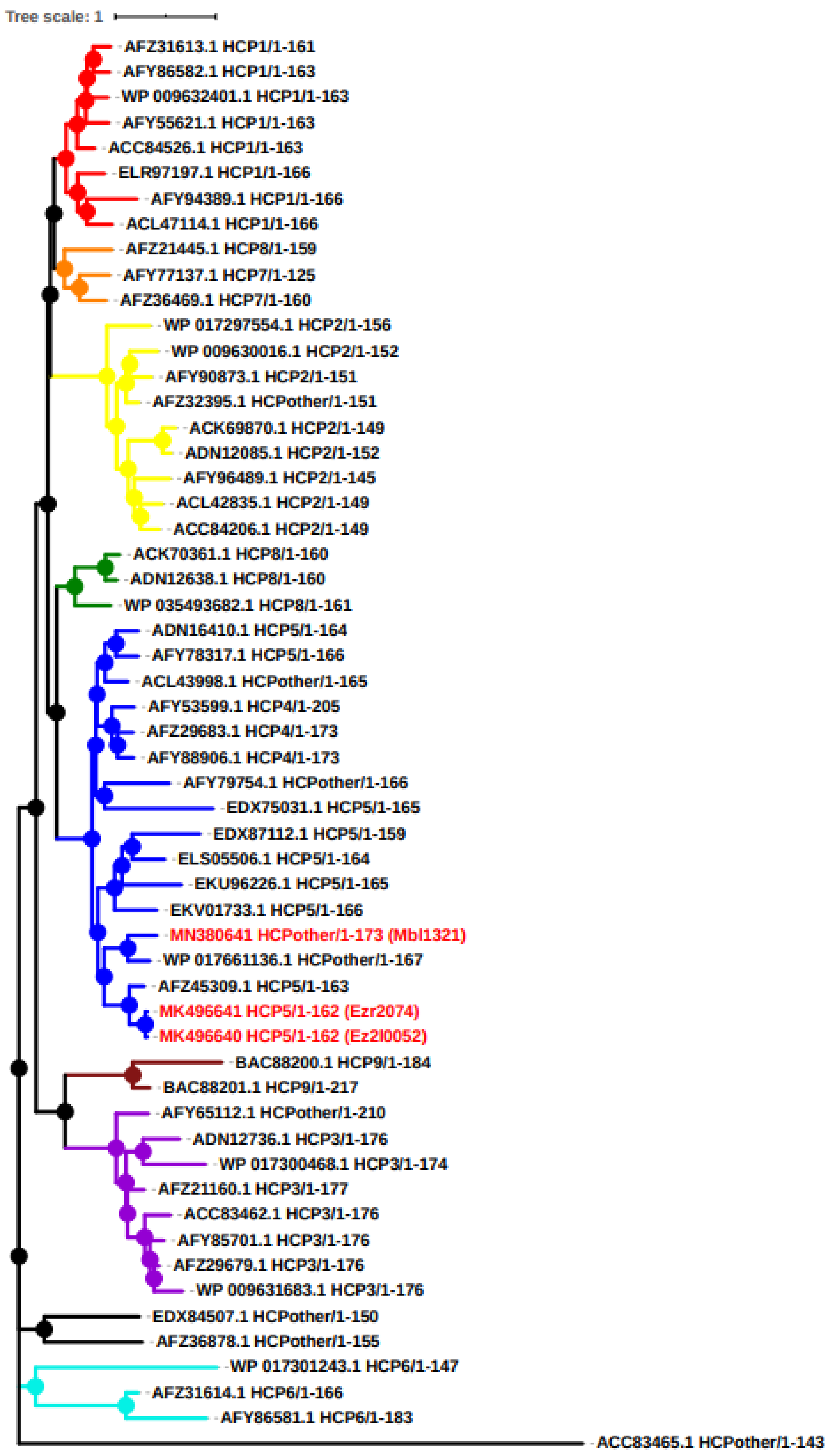

Tree scale: 1
AFZ31613.1 HCP1/1-161
AFY86582.1 HCP1/1-163
WP 009632401.1 HCP1/1-163
AFY55621.1 HCP1/1-163
ACC84526.1 HCP1/1-163
ELR97197.1 HCP1/1-166
AFY94389.1 HCP1/1-166
ACL47114.1 HCP1/1-166
AFZ21445.1 HCP8/1-159
AFY77137.1 HCP7/1-125
AFZ36469.1 HCP7/1-160
WP 017297554.1 HCP2/1-156
WP 009630016.1 HCP2/1-152
AFY90873.1 HCP2/1-151
AFZ32395.1 HCPother/1-151
ACK69870.1 HCP2/1-149
ADN12085.1 HCP2/1-152
AFY96489.1 HCP2/1-145
ACL42835.1 HCP2/1-149
ACC84206.1 HCP2/1-149
ACK70361.1 HCP8/1-160
ADN12638.1 HCP8/1-160
WP 035493682.1 HCP8/1-161
ADN16410.1 HCP5/1-164
AFY78317.1 HCP5/1-166
ACL43998.1 HCPother/1-165
AFY53599.1 HCP4/1-205
AFZ29683.1 HCP4/1-173
AFY88906.1 HCP4/1-173
AFY79754.1 HCPother/1-166
EDX75031.1 HCP5/1-165
EDX87112.1 HCP5/1-159
ELS05506.1 HCP5/1-164
EKU96226.1 HCP5/1-165
EKV01733.1 HCP5/1-166
MN380641 HCPother/1-173 (Mbl1321)
WP 017661136.1 HCPother/1-167
AFZ45309.1 HCP5/1-163
MK496641 HCP5/1-162 (Ezr2074)
MK496640 HCP5/1-162 (Ez2l0052)
BAC88200.1 HCP9/1-184
BAC88201.1 HCP9/1-217
AFY65112.1 HCPother/1-210
ADN12736.1 HCP3/1-176
WP 017300468.1 HCP3/1-174
AFZ21160.1 HCP3/1-177
ACC83462.1 HCP3/1-176
AFY85701.1 HCP3/1-176
AFZ29679.1 HCP3/1-176
WP 009631683.1 HCP3/1-176
EDX84507.1 HCPother/1-150
AFZ36878.1 HCPother/1-155
WP 017301243.1 HCP6/1-147
AFZ31614.1 HCP6/1-166
AFY86581.1 HCP6/1-183
ACC83465.1 HCPother/1-143

C

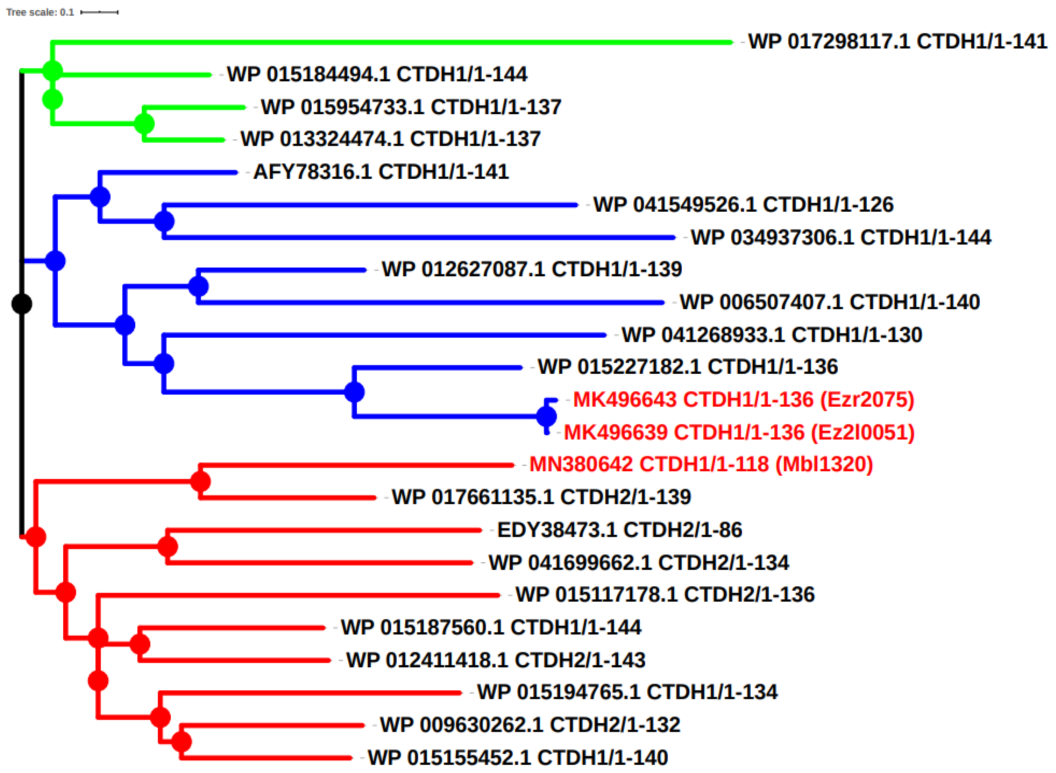


**Supplementary Figure S3. Phylogenetic tree of OCP, HCP, and CTDH.**

(A) Phylogenetic tree of OCPs. OCPs from *Euhalothece* Z-M001 (Ezr1969) and *Microcoleus* B353 (Mbr0566) are colored red, whereas the OCP from *Synechocystis* PCC6803 (Slr1963) is colored blue.

(B) Phylogenetic tree of HCP sequences. HCPs form *Euhalothece* ZM001 (Ezr2074), *Euhalothece* Z9404 (Ez2l0052), and *Microcoleus* B353 (Mbl1321) are indicated in red.

(C) Phylogenetic tree of CTDH sequences. CTDHs from *Euhalothece* Z-M001 (Ezr2075), *Euhalothece* Z9404 (Ez2l0051), and *Microcoleus* B353 (Mbl1320) are indicated in red.

Subclades identified in the phylogenetic trees of OCPs, HCPs, and CTDHs were defined based on previous studies[2-4]. In this study, HCP sequences were grouped into five subclades. Protein sequences used in phylogenetic analysis are listed in Supplementary Table S1.

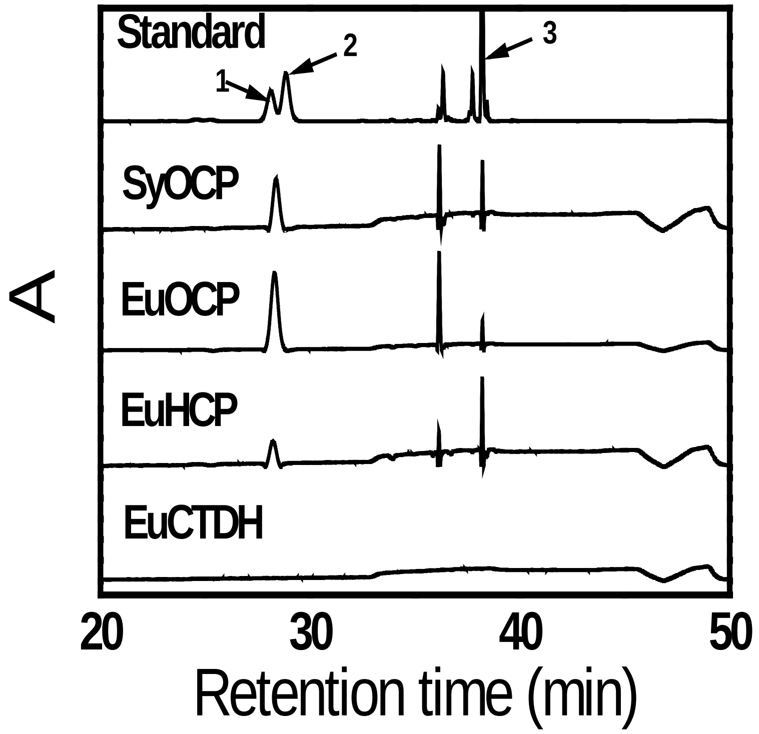


**Supplementary Figure S4. HPLC analysis of EuOCP-, EuHCP-, and EuCTDH-bound carotenoids purified from Zea-producing *E. coli*.** 1, Zea; 2, Can; 3, β-carotene.

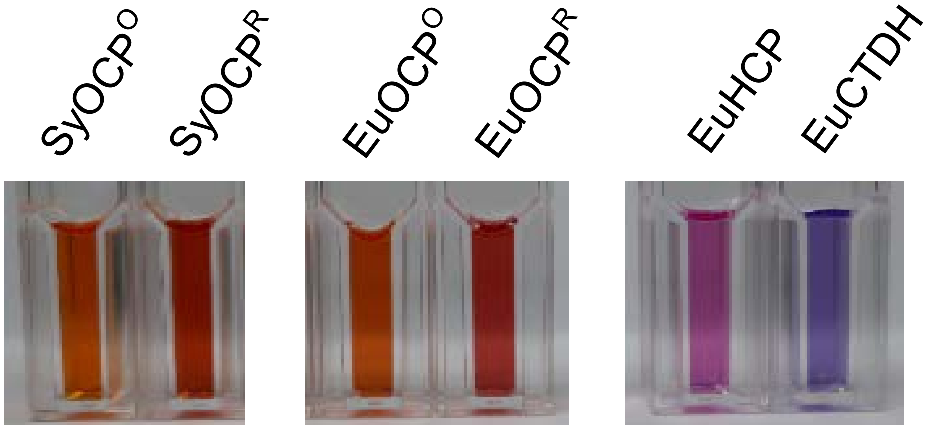


**Supplementary Figure S5. Purified OCPs and their paralogs (HCPs and CTDHs) isolated from Can-producing *E. coli*.**

Sy, *Synechocystis*; Eu, *Euhalothece*. Orange indicates OCPs before exposure to light (OCP$^{O}$), and red lines represent OCPs after exposure to light (OCP$^{R}$). EuHCP and EuCTDH were non-responsive to illumination.
